# Clinical Perspectives on the Injectability of Cross-Linked Hyaluronic Acid Dermal Fillers: A Standardized Methodology for Commercial Product Benchmarking with Inter-Injector Assessments

**DOI:** 10.3390/gels10020101

**Published:** 2024-01-26

**Authors:** Patrick Micheels, Alexandre Porcello, Thierry Bezzola, Daniel Perrenoud, Pierre Quinodoz, Yogeshvar Kalia, Eric Allémann, Alexis Laurent, Olivier Jordan

**Affiliations:** 1 Private Medical Practice, CH-1224 Chêne-Bougeries, Switzerland; 2 School of Pharmaceutical Sciences, University of Geneva, CH-1211 Geneva, Switzerland; alexandre.porcello@unige.ch (A.P.); yogi.kalia@unige.ch (Y.K.); eric.allemann@unige.ch (E.A.); olivier.jordan@unige.ch (O.J.); 3 Institute of Pharmaceutical Sciences of Western Switzerland, University of Geneva, CH-1211 Geneva, Switzerland; 4 Private Medical Practice, CH-1204 Geneva, Switzerland; tbezzola@gmail.com; 5 Private Medical Practice, CH-1006 Lausanne, Switzerland; drperrenoud@gmail.com; 6 Hôpital de la Tour, CH-1217 Meyrin, Switzerland; pierre.quinodoz@latour.ch; 7 Regenerative Therapy Unit, Lausanne University Hospital, University of Lausanne, CH-1015 Lausanne, Switzerland; alexis.laurent@unil.ch; 8 Manufacturing Department, TEC-PHARMA SA, CH-1038 Bercher, Switzerland; 9 Manufacturing Department, LAM Biotechnologies SA, CH-1066 Epalinges, Switzerland

**Keywords:** aesthetic medicine, cross-linked hyaluronic acid, dermal fillers, ex vivo skin, hydrogel systems, injectability, lidocaine, medical device, needle gauge, product benchmarking

## Abstract

The injectability of cross-linked hyaluronic acid (HA) dermal fillers is influenced by polymer concentration, polymer cross-linking type and degree, the presence of lidocaine or other functional excipients, types of syringes, and injection techniques. Finished product injectability constitutes a critical quality attribute for clinical injectors, as it strongly influences product applicability and ease of use in aesthetic medicine. While injectable product extrusion force specifications are provided by the respective device manufacturers, the qualitative informative value of such datasets is low for injectors wishing to compare product brands and technologies from an injectability standpoint. Therefore, the present study comparatively assessed 28 cross-linked HA dermal fillers (JUVÉDERM^®^, Restylane^®^, BELOTERO^®^, TEOSYAL RHA^®^, and STYLAGE^®^ brands) using various injectability benchmarking setups for enhanced clinical-oriented relevance. Manual product injections were performed by three specialized and experienced clinicians, whereas automatic product extrusion was performed using a Texture Analyzer instrument. The various hydrogel products were injected into ex vivo human skin and into SimSkin^®^ cutaneous equivalents to appropriately account for injection-related counterpressure. The injectability results revealed important variability between and within product brands, with a strong influence of the local anesthetic lidocaine, HA contents, and needle gauge size. Critical appraisals of the investigated products were performed, notably from manufacturing process-based and clinical ease of application-based standpoints, centered on respective experimental injectability quality levels. Generally, it was confirmed that each HA-based dermal filler product requires specific expertise for optimal injection, mainly due to differing viscoelastic characteristics and injectability attributes. Overall, the present study set forth evidence-based and clinical-oriented rationale elements confirming the importance for injectors to work with injectable products with which they are experienced and comfortable to optimize clinical results.

## 1. Introduction

Dermal filler injections are nonsurgical cosmetic procedures that are used to plump up wrinkles, smooth cutaneous lines and/or creases and restore fine volumes in the face [1]. Hyaluronic acid (HA)-based dermal fillers stand out at the upper end of the quality spectrum [2]. This is due to their documented safety, efficacy, and reversibility, with more than 2.6 million procedures performed in the USA alone in 2020 [3,4]. Modern HA-based dermal fillers each exhibit specific properties owing to different cross-linking technologies, product compositions, and manufacturing processes [5,6,7,8]. The primary objective of manufacturer-specific formulation and production technologies is to adjust the finished product’s mechanical or biophysical properties. Such attributes may be finely tuned to closely mimic the biological tissues surrounding the administration site, enabling positive post-injection physiological outcomes [9,10,11]. Despite the invasive nature of dermal filler administration via localized injection, reported complication rates are low in the hands of medical professionals [12,13,14].

Among the important biophysical attributes of HA-based dermal fillers, system viscoelasticity, cohesivity, water uptake, and injectability are often cited [6,7,8,14,15]. Dynamic effects pertaining to product stability and biodistribution are also important from a product development standpoint [16,17,18]. In addition, specific clinical-oriented product characterization should consider the behavior of the hydrogel system in the primary packaging elements during administration. Throughout the injection process, the hydrogel within the syringes undergoes a variety of stresses and forces. In further detail, the gels are exposed to shear stress, along with vertical compression or elongation forces, resulting in material deformation. Therein, under minimal stress, homogeneous dermal fillers tend to approach the behavior of purely gel-like substances. As the shear stress intensifies, the system exhibits fluid-like behaviors and starts to flow, enabling extrusion through the needle [14]. Of note, the flow resistance is then influenced by external parameters, namely the cutaneous layer in which the gel is implanted, which generates counterpressure. Therein, the subcutaneous tissue generally presents less resistance, as it presents lobules between which HA-based gels can permeate without damaging the cells. Conversely, the dermis is predominantly fibrous, and product distribution and dispersion are influenced by gel cohesivity attributes [17,19].

Upon reviewing the clinical practices around HA-based dermal filler injections in aesthetic medicine, high diversity may be observed. Notably, while some injections are performed in the dermis, others are performed directly into the subcutaneous tissue [20]. Furthermore, the injection techniques vary among practitioners, ranging from closely spaced point-by-point injections to retro-tracing and bolus injections. The latter is especially used for subcutaneous tissue supplementation and in proximity of osseous matter for volumetric correction. Additionally, injectors may use the thumb pad, the thumb’s interphalangeal joint, or the thenar eminence to actuate the syringe. This diversity is best exemplified among mesotherapy practitioners [21].

Overall, the extent to which HA-based dermal fillers flow largely depends on their manufacturing conditions, composition, the method and speed of injection, and the injection site. The diversity in available products and the multiple parameters influencing clinical outcomes highlight the importance of evidence-based dermal filler product selection for specific aesthetic applications. As regards product quality attributes, an ideal dermal filler should be easy to inject, with homogeneous (i.e., within one product unit and between product lots) and consistent product behavior upon administration. This ensures optimal performance by conferring the highest level of control over the product and the administration process, which in turn contributes to minimizing administration-related discomfort [14]. The latter may be mitigated by specific formulation means, such as the incorporation of local anesthetics (e.g., lidocaine). Therein, the ancillary pharmacological action of lidocaine may address the administration-related pain stimuli and early patient local reactions.

Since the commercial introduction of non-cohesive HA-based gels in 1994, the practicing co-authors have personally experienced fluctuations in the pressure exerted on dermal filler syringes, particularly during injections using the “Blanching technique” [22,23,24]. Of note, product manufacturers specify forces of ejection and extrusion curves, which are obtained in the laboratory with hydrogel extrusion in the air. Such data are of low translational relevance, as biological tissues oppose counterpressure upon clinical injection, which depends on the anatomical site and injection depth. Furthermore, cutaneous structures present varying epidermal and dermal proportions, where the dermis is characterized by inhomogeneous density or degrees of dermatoporosis [17,21,25,26,27,28,29].

Instructions for use generally specify that cross-linked HA-based dermal fillers should be injected into the superficial, medium, and deep dermis, in the hypodermis, or in the subperiosteal space [30,31,32,33,34]. Regular personal feedback about perceived in-use variations, either in product viscosity (i.e., both intra- and inter-syringes or lots) or in the required injection force, have been shared with product manufacturers (i.e., Patrick Micheels, unpublished communications, 2000–2023). It is notable that products from the BELOTERO^®^ brand (i.e., original presentation before the addition of lidocaine) could be injected into the superficial reticular dermis [23,24]. After having previously experimentally compared product viscoelastic properties with or without lidocaine, a new area of investigation was designed around the assessment of the pressures exerted on the plunger rods of various cross-linked HA-based dermal fillers present on the European and North American markets [26,27].

Therefore, the purpose of this study was to comparatively assess 28 different cross-linked HA-based dermal fillers (i.e., from the JUVÉDERM^®^, Restylane^®^, BELOTERO^®^, TEOSYAL RHA^®^, and STYLAGE^®^ brands), using various injectability benchmarking setups. The objective of the study was to obtain robust datasets on the injectability attributes of the considered commercial products for enhanced clinical-oriented relevance as compared to available extrusion force information. The novelty and originality of the study consisted of the adopted methodology (i.e., multifaceted and complementary injectability attribute characterization with high translational relevance) and the vast scope of commercial products (*n* = 28), which were benchmarked. The study investigated the primary hypothesis that intra-product and inter-product variability exists as regards injectability when using appropriate cutaneous scaffolds. The study investigated the secondary hypothesis that inter-injector variability exists as regards product injectability. The study also investigated the secondary hypothesis that the incorporation of lidocaine significantly impacts product injectability from quantitative and qualitative standpoints. Overall, this study covered evidence-based and clinical-oriented rationale elements supporting the use of well-characterized and high-quality dermal filler products for clinical proficiency maximization and clinical result systematic enhancement.

## 2. Results and Discussion

### 2.1. Technical Benchmarking of Dermal Filler Product Parameters and Specificities

The modern landscape of HA-based dermal fillers is dense and populated by arrays of diverse injectable products. For the needs of the present study, 28 different commercially available and clinically implemented HA-based dermal filler products were retained and compared in terms of injectability. The predetermined methodology for injectable product selection and inclusion in the study was specified. Namely, widely adopted cross-linked HA-based dermal filler products, commercialized on the European and/or USA markets, were retained. The specific product inclusion criteria in the experimental study were as follows:

- Injectable dermal filler products based on BDDE-cross-linked HA;
- Products from well-established manufacturers with a brand presence on the market >10 years;
- Commercial dermal filler products with a CE mark and/or FDA approval (i.e., medical devices);
- Products indicated for injection in the face for managing fine to medium wrinkles and folds, and for volumizing purposes;
- Products well known and regularly injected by one or more of the practicing co-authors;
- Products available on the European and/or North American markets, with frequent clinical application in Switzerland (i.e., area of practice of the co-authors).

It may be noted here that alternative commercial products conformed to the specified inclusion criteria but were not included in the present study based on co-author consensus. In detail, a compromise was made between a relatively large product panel (i.e., 28 products) and the vast extent of the technical investigations carried out herein on the same products. The 28 products, selected among those proposed by five different industrial manufacturers under well-established brand names, were included in the study (Table 1).

Prior to the experimental benchmarking of the retained medical devices (MD) with a focus on injectability attributes, the relevant ad hoc technical documentation was gathered for an initial product technical specification comparison (Table 2).

Furthermore, the retained products were briefly compared in terms of physical, chemical, and rheological attributes for optimal technical description prior to focused injectability attribute determination. The relevant data were gathered from manufacturer-provided documentation or from the scientific literature and are presented in Table S1. While important differences were outlined in terms of rheological properties between the investigated products, major technical and chemical similarities were noted (e.g., BDDE cross-linking, Table S1). The presence of lidocaine in the majority of the investigated dermal fillers justified the specific experimental focus on this component in the subsequent assays (Table 2). Furthermore, the technical investigations around the impact of lidocaine on product injectability attributes were devised based on existing (i.e., incomplete) knowledge of the effects of lidocaine on hydrogel system viscoelasticity [5,6,8].

Despite strong similarities in clinical indications and ingredient compositions (i.e., injection-grade HA), the compared dermal filler products are characterized by specific formulation-related attributes (Table 2). In particular, manufacturer-specific cross-linking technologies constitute one of the major factors of differentiation between product brands, as they notably influence hydrogel biophysical attributes, in vivo product behavior, and in vivo product degradation behavior. However, the exact mechanism for a given cross-linking technology is considered a trade secret. Of note, product manufacturers are required to provide the concentration of HA but not the degree of cross-linking (Table 2).

Interestingly, the degree of cross-linking can influence various critical physicochemical parameters of a product, including its rheology and injectability. Namely, the more a hydrogel system is cross-linked, the higher its elasticity and viscosity will be, making it more challenging to inject. Since the exact degrees of cross-linking are unknown, it is difficult to predict injectability attributes within a given product selection. Within the same range, products can vary in their HA concentration and degree of cross-linking despite using the same cross-linking technology. Of note, 1,4-butanediol diglycidyl ether (BDDE) is the cross-linker used within the HA-based dermal fillers included in the study (Table 2). Notably, BDDE was the first cross-linker to be used in a commercially available dermal filler. Furthermore, this cross-linking type is the one used in most clinical studies, making it the default gold standard [35,36,37,38]. Notwithstanding the high diversity in product offerings outlined in the study, all of the retained product brands and formulation technologies were confirmed to be of current relevance in the field of aesthetic dermatology (Table 2). Specifically, the listed products are available and routinely clinically applied in the accredited clinical centers of the practicing co-authors in Western Switzerland.

### 2.2. Comparative Manual Injectability Evaluation in Ex Vivo Human Skin and in SimSkin^®^ Cutaneous Equivalents

**Figure 1:**
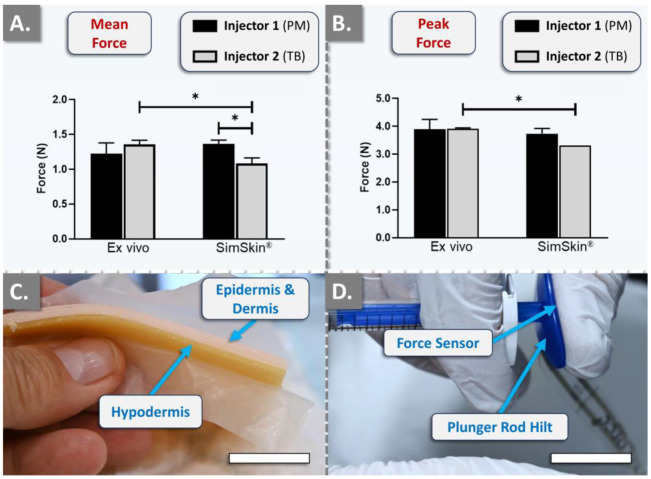
Results of technical equivalence studies for standardized manual hydrogel injectability evaluation, comparing ex vivo human skin and the artificial SimSkin^®^ cutaneous equivalent. The quantitative measurements were performed using the TEOSYAL RHA^®^ 2 product and the FlexiForce^®^ dynamometric sensor attached to the syringe plunger rod hilt. The injections were performed by two clinicians using a point-by-point administration method. (**A**) Quantitative results of the mean injection force. (**B**) Quantitative results of the maximum peak force. Experiments were performed in triplicate, and the results were presented as average values assorted to corresponding standard deviations as error bars. Statistically significant differences (i.e., * or *p*-value < 0.05) were found between the average values. Detailed results of the statistical analyses are presented in Table S2. (**C**) Profile view of the SimSkin^®^ cutaneous equivalent. Scale bar = 10 mm. (**D**) Profile view of the manual injection setup featuring the FlexiForce^®^ dynamometric sensor, positioned beneath the thumb of the injector on the engaged plunger rod hilt. Scale bar = 15 mm. PM, Patrick Micheels; TB, Thierry Bezzola.

In order to reduce the biological variability linked to the use of human skin for the subsequent large-scale comparative hydrogel product injectability studies, a standardized cutaneous equivalent was procured. For the initial validation of the SimSkin^®^ in vitro injectability setup, comparative quantitative analyses were performed against ex vivo human skin for the assessment of potential technical equivalence. A wrinkle-filling product (i.e., TEOSYAL RHA^®^ 2, Teoxane, Geneva, Switzerland) was retained for the experiments and was manually and sequentially injected point-by-point into the superficial to medium dermis of the ex vivo scaffolds and in the superficial polymer layer of the in vitro model. The results were expressed as mean forces of injection and as maximum peak forces of injection (Figure 1).

For both the in vitro and the ex vivo setup, the needle was inserted tangentially into the skin plane (i.e., at an angle < 10°). The results revealed no statistically significant differences between injectors for the mean force and the maximum peak force of injection in the ex vivo skin group (Figure 1A,B). Furthermore, no statistically significant differences were observed for injector 1 (PM) between the ex vivo and the in vitro groups (Figure 1A,B). Conversely, the results obtained for injector 2 (TB) showed statistically significant differences between the ex vivo and the in vitro groups, with a slight decrease in mean forces (−20%) and maximum peak forces (−15%) of injection when TEOSYAL RHA^®^ 2 was injected into SimSkin^®^ (Figure 1A,B). Such different behavior between the injectors was attributed mostly to anatomical specificities of the thumb of the injectors and of the ex vivo skin samples, confirming the need for setup standardization and force measurement normalization. Notably, no very statistically significant or extremely statistically significant differences (i.e., *p*-values < 0.01) were observed between the groups and between the injectors (Figure 1). While the equivalence between the ex vivo and in vitro skin substrates could not be experimentally validated, the need for setup standardization and the absence of very statistically significant differences warranted the further use of SimSkin^®^ cutaneous equivalents for all subsequent injectability studies. Specifically, the recorded standard deviation values in the SimSkin^®^ setup ranged from almost zero to 20% in the experimental trials, which was assessed as satisfactory given the inter- and intra-sample variability of ex vivo cutaneous models.

### 2.3. Inter-Injector Variability Assessment and Influence of Lidocaine on Product Manual Injectability

**Figure 2:**
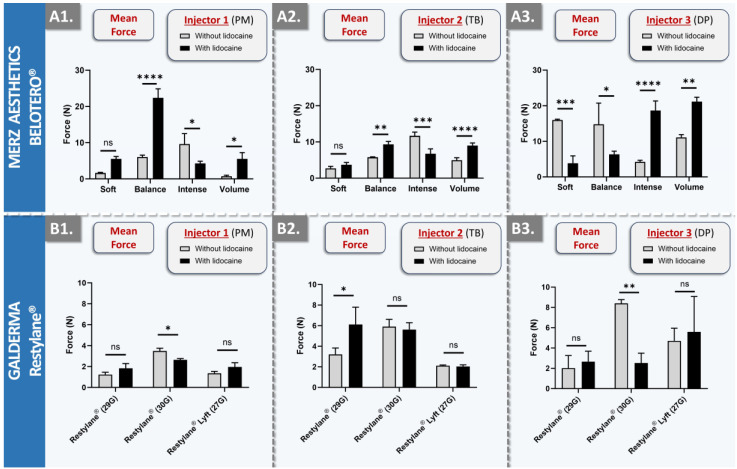
Results of in vitro manual injectability studies for the assessment of inter-injector variability and quantification of the influence of lidocaine on product injectability. The quantitative measurements were performed using the SimSkin^®^ cutaneous equivalent and the FlexiForce^®^ dynamometric sensor. The injections were performed using a point-by-point administration method. (**A1**–**A3**) Comparison of mean injection forces for BELOTERO^®^ products (i.e., with and without lidocaine) between the three injectors. (**B1**–**B3**) Comparison of mean injection forces for Restylane^®^ products (i.e., with and without lidocaine) between the three injectors. Experiments were performed in triplicate, and results were presented as average values assorted to corresponding standard deviations as error bars. Statistically significant differences (i.e., * or *p*-value < 0.05), very significant differences (i.e., ** or 0.001 < *p*-value < 0.01), or extremely significant differences (i.e., *** or 0.0001 < *p*-value < 0.001; **** or *p*-value < 0.0001) were found between the average values. Detailed results of the statistical analyses are presented in Table S3. DP, Daniel Perrenoud; G, gauge; ns, non-significant; PM, Patrick Micheels; TB, Thierry Bezzola.

In order to robustly assess the influence of lidocaine on dermal filler product injectability attributes, several BELOTERO^®^ and Restylane^®^ products (i.e., variants with and without lidocaine) were injected by three clinicians into SimSkin^®^ cutaneous equivalents, using a point-by-point injection technique (Figure 2).

For all injections, the needle was inserted tangentially into the skin plane (i.e., at an angle < 10°). As concerns the injectability of the dermal fillers from the BELOTERO^®^ brand, the results revealed important inter-product and inter-injector diversity (Figure 2(A1–A3)). While lidocaine presence in the hydrogel system generally resulted in significant injectability attribute modulation, no clear trend was outlined across the board. Of note, the behavior of the Balance, Intense, and Volume products with lidocaine for point-by-point injections was found to be significantly different compared to the product variant without lidocaine. While mean forces of the Balance product were significantly higher with lidocaine for injectors PM and TB, they were found to be inferior for injector DP (Figure 2(A1–A3)). Of further note, the presence of lidocaine in the Volume product systematically resulted in higher mean forces for all three injectors (Figure 2(A1–A3)). It was generally observed that the spread between minimal and maximal force values within the product brand was injector-specific (e.g., low overall spread for injector TB, high spread for injector DP; Figure 2(A1–A3)). Finally, it was observed that the recorded force values vary depending on the injection speed and the area of the thumb used to apply pressure on the plunger rod hilt (Table S4). Specifically, higher values were recorded when the pressure was applied through the P1-P2 joint of the thumb, as opposed to the thumb pad (Figure S1).

As concerns the injectability of the dermal fillers from the Restylane^®^ brand, the result trends were comparable to those of the BELOTERO^®^ brand in terms of the influence of lidocaine presence, intra-brand behavior, and inter-injector behavior (Figure 2). A major difference between the two brands was noted in terms of force scale, where mean values were found to be inferior across the board for the Restylane^®^ products (Figure 2). Additionally, the force differential between product variants (i.e., with or without lidocaine) was generally found to be lower for the Restylane^®^ products as compared to the BELOTERO^®^ products (i.e., lower levels of statistical significance; Figure 2). Specifically considering the Restylane^®^ brand, a trend of higher mean injection force values was outlined for higher-gauge needles, as expected (Figure 2(B1–B3)). From a formulation viewpoint, it is notable that the NASHA^®^ technology used in the manufacture of Restylane^®^ gels is different from the CPM^®^ technology of BELOTERO^®^ gels (Table 2). Furthermore, Restylane^®^ products share a constant concentration of 20 mg/mL HA, while BELOTERO^®^ products contain HA concentrations ranging from 20 mg/mL to 26 mg/mL (Table 2). No correlation was found between the concentration of the HA-based polymer and the mean force of injection of the product. Additionally, according to the literature, the degree of substitution of Restylane^®^ is around 1.2%, whereas no information can easily be found for the CPM^®^ technology [39,40,41].

The viscosity and the cohesivity (i.e., measurement of maximum normal forces) of Restylane^®^ products were lower than the viscosities of BELOTERO^®^ products, except for the Soft variant, which was found to be congruent with the respective mean forces of injection, especially for injectors 1 and 2 [42,43]. Such quantitative elements may partly explain the lower overall mean injection force values recorded for the Restylane^®^ products (Figure 2 and Table S5). Finally, the lowest overall injection force value spread for Restylane^®^ products was identified for injector PM (Figure 2(B1)). Importantly, it was recorded that injector PM routinely administers Restylane^®^ products, whereas injectors TB and DP do not. Therefore, it may be assessed that a learning phase exists for products that are not routinely administered (i.e., even by specialized physicians) and that practice and experience may result in diminished administration-related variability. Moreover, the investigational panel of three injectors is diverse, consisting of a general practitioner, a surgeon, and a dermatologist. These three injectors do not share the same injection habits. Injector 3 typically injects very small volumes and may prick a patient’s face up to a hundred times. However, this injector administers dermal fillers to an average of 10 patients per week. Injectors 1 and 2, on the other hand, treat well over 10 patients per week with fillers. It is also noteworthy that injector 1 tends to inject at a slower pace compared to injectors 2 and 3.

**Figure 3:**
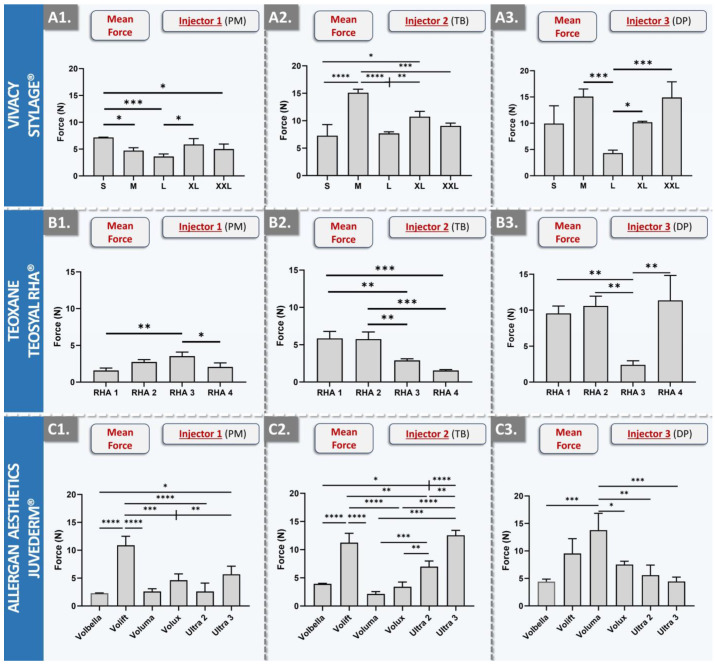
Results of in vitro manual injectability studies for the assessment of inter-injector variability over a diverse panel of dermal filler products. The quantitative measurements were performed using the SimSkin^®^ cutaneous equivalent and the FlexiForce^®^ dynamometric sensor. (**A1**–**A3**) Comparison of mean injection forces for STYLAGE^®^ products between the three injectors. (**B1**–**B3**) Comparison of mean injection forces for TEOSYAL RHA^®^ products between the three injectors. (**C1**–**C3**) Comparison of mean injection forces for JUVÉDERM^®^ products between the three injectors. Experiments were performed in triplicate, and the results were presented as average values assorted to corresponding standard deviations as error bars. Statistically significant differences (i.e., * or *p*-value < 0.05), very significant differences (i.e., ** or 0.001 < *p*-value < 0.01), or extremely significant differences (i.e., *** or 0.0001 < *p*-value < 0.001; **** or *p*-value < 0.0001) were found between the average values. Detailed results of the statistical analyses are presented in Table S3. DP, Daniel Perrenoud; PM, Patrick Micheels; TB, Thierry Bezzola.

As concerns the injectability of the dermal fillers from the STYLAGE^®^ brand, the results displayed the lowest overall intra-brand and inter-injector variability (Figure 3(A1–A3) and Table S6).

Similar to the results of the Restylane^®^ product brand, the lowest intra-brand spread in force values was recorded for the injector PM (Figure 3(A1)). Furthermore, the lowest overall mean injection force values for the STYLAGE^®^ brand were also recorded for injector PM (Figure 3(A1–A3)). Specifically, STYLAGE^®^ S required the greatest mean injection force for injector PM, with 1.68 N. STYLAGE^®^ M, XL, and XXL necessitated intermediate forces of 1.11 N, 1.38 N, and 1.18 N, respectively, demonstrating marginal variations within the brand. The lowest required force was recorded for STYLAGE^®^ L, averaging at 0.78 N (Figure 3(A1)). Of note, STYLAGE^®^ XXL is the only product variant without lidocaine (Figure 3(A1–A3) and Table 2). While STYLAGE^®^ products differ primarily in their intended uses, needle size, and HA concentration, they all employ the same IPN-Like^®^ cross-linking technology and mannitol as an integrated antioxidant (Table 2) [44,45]. The relevant literature shows that STYLAGE^®^ M displays an injection force close to 10 N at a speed of 13 mm·min^−1^, which represents an appropriate level for manual injection [46]. The analysis of the experimental datasets revealed that injectors 2 and 3 recorded slightly higher forces, whereas injector 1 recorded slightly lower forces. Notwithstanding, all values were found to fall within a comparable range, and the lower results for injector 1 could be linked to slower speeds of injection in comparison with injectors 2 and 3 [46].

As concerns the injectability of the dermal fillers from the TEOSYAL RHA^®^ brand, the results displayed intra-brand injection force variability and inter-injector force scale differences (Figure 3(B1–B3) and Table S7). Specifically, brand overall mean injection forces were found to be relatively low for injector PM, intermediate for injector TB, and high for injector DP (Figure 3(B1–B3)). The TEOSYAL RHA^®^ products all employ the same Preserved Network^®^ cross-linking technology and contain lidocaine, differing primarily in their intended uses, needle size, and HA concentration (Table 2) [34,47]. From a rheological viewpoint, RHA^®^ 1 exhibits the lowest values, RHA^®^ 2 and 3 score in the same range, and RHA^®^ 4 scores the highest in value [47]. For injector PM, the exerted force increased from RHA^®^ 1 to RHA^®^ 3 (i.e., from 0.37 N to 0.83 N) and then decreased for RHA^®^ 4 (i.e., 0.49 N; Figure 3(B1)). Such patterns may be rationally explained by the specific HA concentrations and the intended product uses (Table 2). In detail, products designed for treating deep folds and for adding volume (e.g., RHA^®^ 3) typically comport a higher HA concentration, thus requiring superior injection forces. RHA^®^ 3 exhibited lower values than those of RHA^®^ 2 for injectors 2 and 3, and this was explained by the fact that RHA^®^ 2 and RHA^®^ 3 show rheological values in the same range, but where RHA^®^ 3 was injected with a 27 G needle (i.e., while RHA^®^ 2 was used with a 30 G needle) [47]. Interestingly, the brand-wide trend of injection forces was inverted for injector TB as compared to injector PM, where RHA^®^ 1 and RHA^®^ 2 scored the highest (Figure 3(B1,B2)). Such results clearly indicated differences in the specific injection techniques used by each injector.

As concerns the injectability of the dermal fillers from the JUVÉDERM^®^ brand, the results outlined high intra-brand variability but the lowest overall inter-injector variability (Figure 3(C1–C3) and Table S8). In detail, the results generally indicated again that injectors TB and DP typically apply more force than injector PM (i.e., with the exception of the Volux and Voluma products, when comparing injectors TB and PM, Figure 3(C1–C3)). As for the other investigated product brands, the recorded differences in the required injection forces are most likely a composite effect of several variables, including the HA concentration, needle gauge, and the specific cross-linking technology (Figure 2 and Figure 3, Table 2). Interestingly, Volbella and Volift are the two products that were assessed as being consistent in terms of injection force for the three injectors, corresponding to the products with the lower HA concentrations within the JUVÉDERM^®^ brand. From a rheological viewpoint, concerning the hydrogels using the VYCROSS^®^ technology, Volbella displayed the lowest injectability values and Volux the highest. Of note, the Hylacross^®^ technology used in the JUVÉDERM^®^ brand shows higher values of tan delta (i.e., ratio of storage modulus/loss modulus), suggesting a lower cross-linking percentage in comparison with the VYCROSS^®^ technology [43]. With regard to the important inter-injector differences that were recorded across the retained product panel, most may be attributed to the specific product administration technique and the respective experience levels of any given injector with a specific product. Importantly, such findings underscore the importance of conjoint injector and product selection, as different combinations were shown herein to significantly influence the required injection force (Figure 2 and Figure 3). As the latter may potentially impact the ease of product injection, such choices may prove to be determinants in the overall patient experience [48,49].

### 2.4. Automated In Vitro Product Injectability Assessment: Comparative Injection Curves for Standardized Dermal Filler Product Benchmarking

While the previous section (i.e., manual product injections in SimSkin^®^ substrates) enabled to underscore inter-injector diversity and significant impacts of lidocaine presence on product injectability attributes, the assessments of intra-product injectability variability were limited (Tables S3–S7). Specifically, while the injection force was measured during a complete hydrogel unit extrusion cycle (i.e., emptying of the product syringe), the obtained force profile records only enabled to transcribe mean injection forces and peak injection forces (Figure 2 and Figure 3, Tables S3–S7). In order to experimentally investigate the main hypothesis of the study (i.e., intra-product injectability variability), automated in vitro product injectability assessments were performed. This setup enabled normalization of the injection speed for each product and eliminated the potential bias of injector experience with one product brand but not with another.

**Figure 4:**
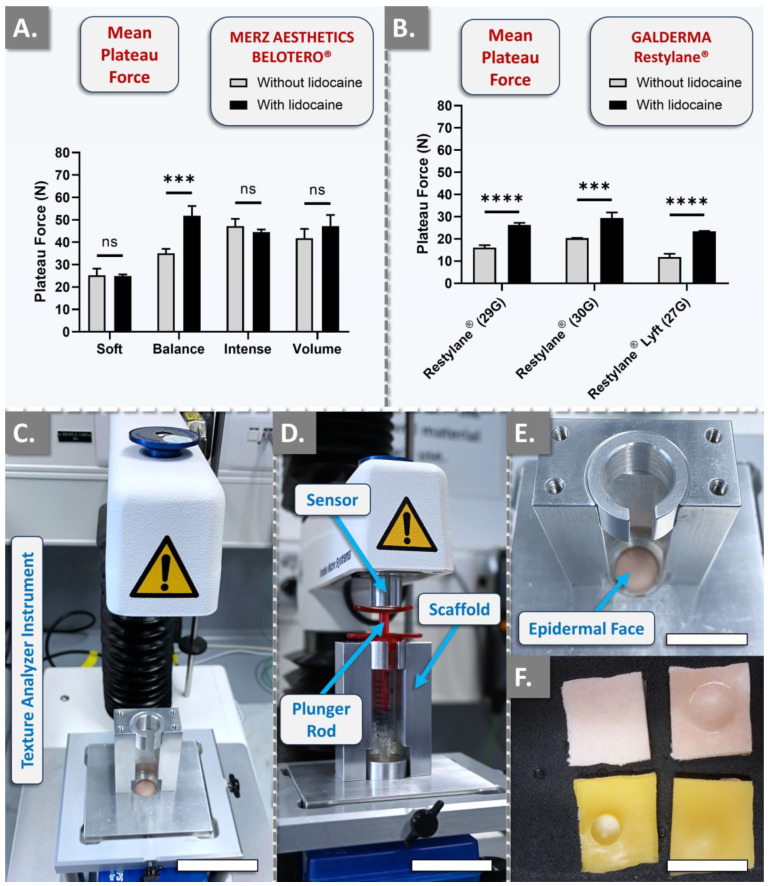
Results of in vitro automated injectability studies for the quantification of the influence of lidocaine on product injectability. The quantitative measurements were performed using a constant plunger rod actuation speed of 1 mm·s^−1^. (**A**) Comparison of mean plateau injection forces for BELOTERO^®^ product variants (i.e., with and without lidocaine). (**B**) Comparison of mean plateau injection forces for Restylane^®^ product variants (i.e., with and without lidocaine). Experiments were performed in triplicate, and the results were presented as average values assorted to corresponding standard deviations as error bars. Extremely significant statistical differences (i.e., *** or 0.0001 < *p*-value < 0.001; **** or *p*-value < 0.0001) were found between the presented mean values. Detailed results of the statistical analyses are presented in Table S9. (**C**) Experimental setup for automated hydrogel injectability measurements. Scale bar = 5 cm. (**D**) Product syringe loaded in the automated injectability measurement setup. Scale bar = 4 cm. (**E**) Top view of the SimSkin^®^ cutaneous equivalent (i.e., epidermal side) in the automated injectability measurement setup. Scale bar = 2 cm. (**F**) Epidermal side (top) and hypodermal side (bottom) of the SimSkin^®^ cutaneous equivalent, before and after hydrogel product injection. Scale bar = 2 cm. ns, non-significant.

The first part of the automated injectability study enabled us to assess once more the impact of lidocaine presence on product injectability attributes [50,51]. The results, which were, respectively, obtained with BELOTERO^®^ and Restylane^®^ variants (i.e., with and without lidocaine), revealed either an absence of statistically significant difference in mean plateau injection forces (i.e., Soft, Intense, and Volume products) or extremely statistically significant differences between the respective variants (Figure 4A,B).

Of note, only the Balance product was found to behave specifically within the BELOTERO^®^ brand, as all other variants were, respectively, not found to behave statistically differently from each other (Figure 4A). In detail, the Balance variant with lidocaine exhibited injection forces 1.5 times higher on average than its lidocaine-free counterpart, corroborating the manual findings of injectors PM and TB during point-by-point injection (Figure 2(A1–A3)). Importantly, the Balance variant with lidocaine was recorded as more homogeneous during manual injection than its lidocaine-free counterpart (Table S4). Consideration of the automated injection curves for the Balance variants corroborated such observations, where the variant with lidocaine presented much smoother plateau injection forces (Figure S2). Moreover, when comparing the results obtained with those of manual injections, it is interesting to note that BELOTERO^®^ Volume (i.e., which indicated higher values for the version with lidocaine for each injector) presented similar trends at a constant speed.

Specific consideration of the Restylane^®^ variants confirmed the systematic and significant increase in mean plateau injection forces in the lidocaine-containing products (Figure 4B). In detail, a two-fold increase was recorded for Restylane^®^ Lyft, a 1.6-fold increase was recorded for Restylane^®^ injected with a 29 G needle and a 1.4-fold increase was recorded for Restylane^®^ administered with a 30 G needle (Figure 4B and Figure S3). It is of great interest to note that there is a lack of studies investigating the interactions between lidocaine and HA, especially when considering the number of products that contain such functional excipients [41,50,51]. Nevertheless, the presented results clearly indicated that most product variants with lidocaine were more difficult to inject than their lidocaine-free counterparts (Figure 4, Figures S2 and S3).

**Figure 5:**
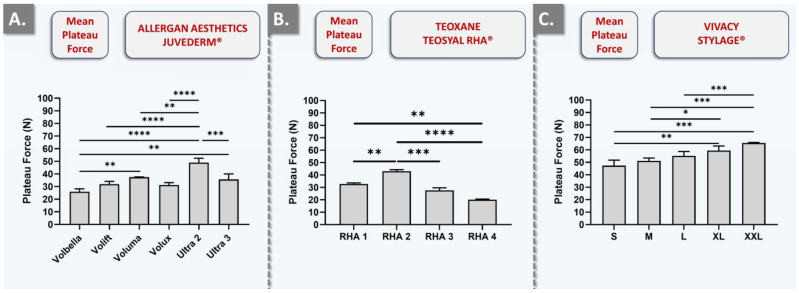
Results of in vitro automated injectability studies for the assessment of dermal filler intra-brand injection force variability. The quantitative measurements were performed using the SimSkin^®^ cutaneous equivalent and a constant plunger rod actuation speed of 1 mm·s^−1^. (**A**) Comparison of the mean plateau injection forces required for JUVÉDERM^®^ products. (**B**) Comparison of the mean plateau injection forces required for TEOSYAL RHA^®^ products. (**C**) Comparison of the mean plateau injection forces required for STYLAGE^®^ products. Experiments were performed in triplicate, and the results were presented as average values assorted to corresponding standard deviations as error bars. Statistically significant differences (i.e., * or *p*-value < 0.05), very significant differences (i.e., ** or 0.001 < *p*-value < 0.01), or extremely significant differences (i.e., *** or 0.0001 < *p*-value < 0.001; **** or *p*-value < 0.0001) were found between the presented mean values. Detailed results of the statistical analyses are presented in Table S9.

As regards the comparison of the injection force values obtained during manual injection and during automated injection, experimental setup-related rationale elements may be presented. Specifically, the average injection forces were notably higher across the board of investigated products when measured with the Texture Analyzer instrument. An explanation for the higher injection forces as measured in the automated setup is related to the speed of injection. Namely, the average speed for point-by-point manual injection was between 10 and 20 mm·min^−1^. In contrast, the automated and constant injection speed was set at 60 mm·min^−1^. For an equal counterpressure (i.e., mediated by hydrogel physical attributes, needle gauge and length, and cutaneous substrate composition), an increased injection speed will increase the plateau injection force. The corroboration of such mechanical principles with the obtained experimental datasets was confirmed for the JUVÉDERM^®^, TEOSYAL RHA^®^, and STYLAGE^®^ brands, where automated injection forces were systematically recorded as superior to manual injection forces (Figure 5 and Figures S4–S6).

Interestingly, only TEOSYAL RHA^®^ 1 exhibited intra-product variability comparable to that of BELOTERO^®^ Balance without lidocaine (Figure S5). Overall, despite the presence of intra-brand statistically significant differences in automated injectability values, the relative differences were assessed as being lower than in the manual injectability measurement setup (Figure 3 and Figure 5). This may partly be explained by the experimental standardization in the automated setup.

From a technical viewpoint, when comparing products administered with the same needle gauge (e.g., Volbella and Volift), it may be assessed that the force plateau generally rises with the increase in HA concentration (Figure 5 and Figure S4, Table 2). This type of force plateau increase may also be observed for TEOSYAL RHA^®^ products (Figure 5 and Figure S5, Table 2). Additionally, the same observation may be made about STYLAGE^®^ products, which presented the highest mean plateau injection forces (Figure 5 and Figure S6, Table 2). Specifically, STYLAGE^®^ products were found to be significantly tougher to inject in the automated setup as compared to TEOSYAL RHA^®^ and JUVÉDERM^®^ products (Figure 5 and Figure S6). This observation, which was not made in the manual setup, could potentially be attributed to the IPN-Like^®^ cross-linking technology or the addition of mannitol (Figure 3, Table 2). Overall, it was also assessed that plateau injection forces do not linearly correlate with the HA concentration or the needle gauge (Figure 4, Figure 5 and Figures S2–S6, Table 2). This indicated that the true injection force is likely conjointly influenced by HA concentration, needle gauge, product formulation, cross-linking type and degree, and the specific product manufacturing technology. Therefore, in conducting comparative product analyses encompassing all three injectors based on the values acquired via the dynamometric sensor, one encounters substantial challenges. This complexity arises due to the multitude of variable factors involved, such as the position of the thumb, the speed of injection, familiarity with the product, and the hydrogel homogeneity within the syringe.

Consideration of the obtained injectability profiles confirmed the hypothesis that significant variations in the intra-product injection force (i.e., and in the felt pressure) during product administration were possible (e.g., BELOTERO^®^ Balance, TEOSYAL^®^ Ultra Deep; Figures S2 and S5). The obtained data specifically demonstrated that some hydrogel systems present a higher degree of uniformity in their viscoelastic characteristics, both within a single syringe and intra-brand. An example thereof may be set forth with the comparison of the automated injection force profiles of TEOSYAL RHA^®^ products and STYLAGE^®^ products, the latter presenting smoother force plateaus (Figures S5 and S6). Furthermore, intra-product homogeneity was shown to be impacted in product variants containing lidocaine. In detail, non-homogeneous force plateaus were specifically identified for TEOSYAL RHA^®^ 1, Ultra Deep, Voluma, Ultra 2, BELOTERO^®^ Balance, BELOTERO^®^ Volume (i.e., with and without lidocaine), BELOTERO^®^ Intense (i.e., with and without lidocaine), and Restylane^®^ Lyft (i.e., with and without lidocaine; Figures S2–S6).

From a formulation viewpoint, the hydrogel systems utilizing the CPM^®^ cross-linking technology (i.e., with the exception of BELOTERO^®^ Soft and BELOTERO^®^ Intense) display irregular extrusion curves (Figure S2). More specifically, product variants with lidocaine showed higher plateau extrusion forces compared to their variants without lidocaine (Figure S2). For products manufactured with the NASHA^®^ technology, the most traditional variant in the range (i.e., Restylane^®^) demonstrated the most homogeneous viscoelastic properties (Figure S3). In the JUVÉDERM^®^ brand, except for the Voluma^®^ and Ultra 2 variants, products using the VYCROSS^®^ cross-linking technology present very stable extrusion force curves, indicating fairly homogeneous viscoelastic properties (Figure S4). Generally, excluding RHA^®^ 1, the Preserved Network^®^ technology provides hydrogels with excellent viscoelastic properties and high consistency within a single syringe (Figure S5). Finally, STYLAGE^®^ hydrogels manufactured with the IPN-Like™ cross-linking technology exhibit the smoothest profiles, suggesting homogeneity in their viscoelastic properties (Figure S6). The obtained experimental results confirmed and underscored, from a clinically relevant injectability standpoint, that not all cross-linked HA-based dermal fillers are created equal. Overall, it is most likely that each cross-linking technology necessitates specific clinician experience and expertise for effective in vivo application.

### 2.5. Assessment of the Influence of Lidocaine Incorporation in Cross-Linked HA-Based Hydrogel Systems

As previously mentioned, the nature of the interactions between HA-based polymeric systems and lidocaine, a common dermal filler ingredient, is incompletely understood [41,50]. From a purely compositional standpoint, the presence or absence of lidocaine in a given HA-based hydrogel (i.e., linear or cross-linked polymer, lidocaine quantities usually present in dermal fillers) is insufficient to justify significant variations in system viscoelasticity attributes. A critical approach followed by well-advised dermal filler formulators consists of taking into account the product manufacturing process steps and their respective influence on the individual product components [10,36,37,38]. For example, it is well known that steam sterilization drastically negatively impacts the viscoelastic properties of an HA-based hydrogel system by means of HA chain breakdown. The manufacturing process therefore creates the need for pre-emptive formulation correction, typically with the selection of high molecular weight HA, which accounts for polymer breakdown during product terminal sterilization and eventually enables the reaching of appropriate endpoint molecular weight range technical specifications [52,53].

A similar approach may be used to understand the effects of lidocaine during the manufacturing process of an HA-based dermal filler. For the experimental investigation of such effects, cross-linked HA-based hydrogels with and without lidocaine were prepared and submitted to conservative steam sterilization processing. The endpoint rheological measurement results indicated that the presence of lidocaine during the sterilization step drastically negatively impacted the rheological parameters of the system (i.e., significant additional drop in storage and loss moduli in the lidocaine-containing group; Figure S7). Based on such data, the assumption may be made that the product formulation process or the manufacturing process for dermal filler variants (i.e., same product, with and without lidocaine) must be different if endpoint rheological profiles are similar. Specifically, single or multiple specific technical pre-emptive countermeasures to the negative impacts of lidocaine during sterilization must be employed for products containing lidocaine. This theory is supported by the injectability data of Restylane^®^ variants, where lidocaine-containing products were tougher to inject (Figure 4B).

Although various experimental designs may be used to further investigate the above-mentioned theory on specific process adaptation in the case of lidocaine incorporation, further information about the retained commercial products is not available. Specifically, as each dermal filler product brand is based on different cross-linking technologies and different manufacturing processes, the technical means of lidocaine incorporation may be diverse (Table 2) [54]. Furthermore, as such details on product formulation process and manufacturing specifications are regarded as commercial trade secrets, few product manufacturers would confirm or deny the postulated elements about specific manufacturing-based lidocaine handling precautions. Furthermore, additional research is warranted for the understanding of mechanisms by which lidocaine contributes to additionally or synergistically degrade HA networks during sterilization processing. Such insights could potentially be used to develop new process enhancement options, such as the addition of thermo-protectant agents to lidocaine-containing formulas.

### 2.6. Qualitative Clinical Perspectives for Cross-Linked HA-Based Dermal Filler Administration: Focus on the Point-by-Point Intradermal Injection Technique

Despite the specification of defined intended clinical uses for dermal filler products, clinicians often employ off-label techniques in practice (e.g., different needles and different injection depth) based on their experience. Such off-label administrations may comprise the use of a purely volumizing agent for wrinkle filling in the epidermis or the deeper-than-average use of superficial fillers for enhanced treatment zone plasticity. Using the manual quantitative injectability evaluation setup and point-by-point injections, various off-label administration depths were investigated using the same commercial products and were compared to approved uses in terms of injectability. Injections were performed in SimSkin^®^ substrates by injectors PM and TB.

As concerns the study of JUVÉDERM^®^ products, the point-by-point injection of Volbella into the dermis only required low manual pressures. Conversely, the pressures required to inject Volift in the dermis (i.e., basal portion of the SimSkin^®^ substrate) were high compared to all other HA gels intended for wrinkle filling. Voluma, administered at depths beyond its intended use, necessitated minimal exertion of pressure. Specifically, the gel’s performance during injection was found to be aligned with expectations for a substance that exhibits a high degree of homogeneity in its inherent viscosity. For intradermal administration, the observations for Volux were identical to those for Voluma, namely describing a homogeneous gel requiring low pressures for injection via a 27 G needle. Excluding the Volift product, the hydrogels utilizing the VYCROSS^®^ cross-linking technology generally demonstrated desirable viscoelastic characteristics, and their ease of injectability suggests a uniform gel composition. Regarding the Ultra 2 product, based on the HYLACROSS^®^ cross-linking technology, exceedingly low injection pressures were recorded. Injector TB, operating at a faster pace than injector PM, recorded pressures approximately five times higher with this product. However, as the gel viscosity increased (i.e., using Ultra 3), the differences in applied pressures diminished. Interestingly, injector PM displayed more variation in the exerted pressure than injector TB (i.e., who does not commonly use HYLACROSS^®^ and VYCROSS^®^ gels).

As concerns the study of Restylane^®^ products, the product variant without lidocaine required a very low pressure for intradermal injection with both needle gauge sizes (i.e., 29 G, 30 G). Restylane^®^ without lidocaine is a non-cohesive gel, following the Sundaram-Gavard-Molliard classification [49]. For this product, the 30 G needle is not the manufacturer’s recommended needle. However, the latter was used during the market introduction of the NASHA^®^ gel technology in the late 1990s. In detail, injector PM has maintained the use of this fine needle, which facilitates detailed and precise clinical work. Restylane^®^ Lyft without lidocaine was injected via a 27 G needle and displayed exceptionally low pressures, closely mirroring those observed with the standard Restylane^®^ variant. For Restylane^®^ Lyft with lidocaine, the injection pressures were notably low, albeit slightly superior to those of the lidocaine-free variant, and identical between injectors. Generally, despite being non-cohesive, hydrogels employing the NASHA^®^ cross-linking technology demanded minimal injection pressure for administration into the dermis. However, the addition of lidocaine to the original formulation leads to a minor yet tangible alteration in the properties of the system.

As concerns the study of BELOTERO^®^ products, only the least concentrated and least cross-linked hydrogel (i.e., BELOTERO^®^ Soft) seemed homogeneous in its intrinsic viscosity, as assessed by manual injectability evaluation. Notably, the addition of lidocaine during the manufacture of the Balance product substantially increases the system viscoelasticity, necessitating significantly higher injection pressures. This aspect is particularly noticeable when injecting BELOTERO^®^ Balance via a 30 G needle. Conversely, BELOTERO^®^ Intense, which is designed for treating deep wrinkles (i.e., therefore, theoretically more viscoelastic), necessitated lower injection pressures for the lidocaine-containing variant. Finally, while BELOTERO^®^ Volume is a volumizing agent and therefore expected to display high viscoelasticity, it was easily injected via a 30 G needle. Specifically, the required pressure was systematically low (i.e., for both injectors), regardless of whether or not lidocaine was present.

As concerns the study of TEOSYAL RHA^®^ products utilizing the Preserved Network^®^ cross-linking technology, all investigated products exhibited remarkably low viscoelasticity and easy injectability. Such properties enable effective administration (i.e., including the gel designed for volumetric augmentation) by applying minimal pressure on the syringe plunger rod.

Concerning the study of STYLAGE^®^ products, STYLAGE^®^ S and STYLAGE^®^ M required the highest injection pressure among all the investigated fine wrinkle treatment hydrogels. Nevertheless, it demonstrated excellent intrinsic viscoelastic homogeneity. STYLAGE^®^ XL and XXL are volumizing products and are not recommended for intradermal injection, yet they displayed acceptable pressure levels. Generally, all of the investigated hydrogels utilizing the IPN-Like^®^ cross-linking technology displayed excellent, if not superior, intrinsic viscoelastic homogeneity.

Overall, factors such as the speed of injection, clinician familiarity with the product, and the area of the thumb used to apply pressure on the syringe plunger rod hilt all significantly impact the injectability of dermal fillers. Additionally, it was interesting to observe that during repeated injections (i.e., most often), the injector gains confidence and tends to inject more rapidly, daring to exert higher pressures than during the first injection. Importantly, the specific combination of a skilled injector and a high-quality product plays a crucial role, impacting the ease and effectiveness of the injection process.

### 2.7. Clinical Considerations, Performance Implications, and Perspectives on Product Injectability Attributes

Generally, the experimental results presented herein have confirmed the primary hypothesis of the study as concerns the possible intra-product variability or inhomogeneity in their injectability attributes (Figures S2–S6). Specifically, parallels were made between products with inhomogeneous force injection profiles and the available clinician feedback on injection force discrepancies. This aspect was interpreted to directly support the observations and regular communications of practicing co-author PM on product injectability, for example, in the case of BELOTERO^®^ Balance (i.e., Patrick Micheels, unpublished communications with manufacturers, 2000–2023). Specifically, numerous clinician feedback on injection force inhomogeneity upon administration of BELOTERO^®^ Balance were regularly notified, and such elements were experimentally confirmed by the data presented herein (Figure S2).

The concrete clinical perspectives and significance of the presented data may be approached from a patient safety and intervention quality standpoint. Specifically, in the case of a hydrogel with low homogeneity in its injectability attributes, some irregular indentation-like or jerking behavior of the plunger rod may be felt by the injector during administration. In the worst case, this can potentially lead to variations in the injected gel quantity, where an excess product amount or an inappropriately shallow product injection may be detrimental. Specifically, such administration-related defects may bear tangible consequences for the patient, such as Tyndall effects or the apparition of persistent nodules or lines if too much product is injected superficially [55,56]. Therefore, and importantly, the direct control by the clinician over the administration process of a dermal filler is dependent (i.e., among others) upon product injectability attributes, which may be linked to the safety and effectiveness of the intervention. Overall, as regulatory and reputational damages may be incurred rapidly (i.e., product manufacturers and physicians) in case of in-use safety or efficacy problematics, it is deemed critical for all stakeholders to consider product injectability with an important level of scrutiny.

The injectability data presented herein show that highly significant inter-injector differences may exist in terms of quantitative forces to apply on product syringes. Specifically, this aspect may depend on the experience of the injector with the product, the injection speed, the administration site, or the area of the hand used to inject. Notwithstanding, it may be assessed and set forward that different injection force levels do not necessarily impact the clinical performance of a given homogeneous product. Therein, the most critical factor consists of the in-use level of control of the physician over the dermal filler syringe, which may be correlated with the quality level of the obtained results. Therefore, the mean or peak quantitative aspects of product injectability and the inter-injector variability as concerns injection forces are most probably of lesser importance for clinical performance than the qualitative aspects of product injectability (i.e., intra-product homogeneity, plateau smoothness of injection curves). Such considerations may be of practical use as development perspectives for product manufacturers in particular, which may be urged to fine-tune novel formulas based on quality-driven and clinical-oriented needs.

### 2.8. Study Limitations

The main technical limitation of the present study consisted of the inclusion of only three injectors for the manual product injectability measurements. This specification was linked to the high purchasing costs of the commercial dermal fillers, which were each procured in multiple units. For mitigation of the limited number of injectors, the level of qualification and experience of the retained clinicians with injectable dermal fillers was set high. It should be noted that differences in clinician habits and experience with specific product brands were noted, yet reaching the same levels of clinical experience and ability with all products for all injectors is not tangibly achievable. Such aspects are confirmed by clinical practices in aesthetic medicine, where patient expectation levels are high. Therefore, practitioners generally choose a limited number of products or brands that they master and do not routinely diversify across the board of available commercial products.

A second technical limitation of the present study consisted of the use of different needle gauge sizes for the various injectability experiments. Specifically, strict benchmarking of the 28 retained products in terms of comparative quantitative injectability would have required the use of a single needle reference. However, the choice was made to mainly use the respective needles provided with the dermal fillers in order to conform to manufacturer specifications and to enhance the translational relevance of the obtained datasets.

### 2.9. Future Research Perspectives Based on the Study

Specific future perspectives to the present study comprise in-depth technical assessments of selected products or selected product brands in order to better understand which parameters and specifications influence the quality of the clinical administration process and the quality of the overall patient experience. Therefore, standardized assessments of larger product samples (e.g., evaluating different product lots) would provide enhanced injectability dataset robustness. Furthermore, a specific technical investigation into the formulation-based options for lidocaine incorporation is warranted in order to potentially further optimize specific parameters of product manufacturing. Finally, the datasets presented herein may be used as complements for the clinical education of specialized practitioners. Specifically, the latter classically rely on their own experience with commercial products, yet the majority of the available technical information accompanying a product is manufacturer-provided, which does not allow for the exclusion of various forms of bias.

An additional future perspective to the presented work could also include in vivo injectability pressure measurements during patient treatments. This could potentially facilitate comparative analyses between injections into synthetic cutaneous equivalents and actual facial injections, taking into account the variability in dermal thickness across different treatment areas. Such studies would provide deeper insights into the behavior and properties of these products under real-world application conditions, which could potentially further optimize their use in routine clinical practice.

## 3. Conclusions

The present study provided comprehensive injectability assessments and multi-level comparisons of various dermal filler products, each presenting specificities designed to address different clinical needs. Experimental injectability results revealed important variability between and within product brands, with a strong influence of lidocaine, HA contents, and needle gauge size. Specifically, it was shown that the plateau injection force for cross-linked HA-based dermal fillers varies significantly between brands and even within brands. Notably, this force was not found to correlate linearly with the HA concentration or needle gauge. Critical appraisals of the investigated products were centered on respective experimental injectability quality levels. Intra-product inhomogeneity in terms of exerted pressure during automatic injection was observed notably in systems using NASHA^®^ and CPM^®^ technologies. Conversely, hydrogels using the IPN-Like^®^ cross-linking technology exhibited good homogeneity in their injection profile. Gels containing lidocaine generally displayed conserved or higher injection force trends compared to their counterparts without lidocaine. Generally, it was confirmed that each cross-linked HA-based dermal filler brand and individual product requires specific expertise for optimal injection, mainly due to differing viscoelastic characteristics and specific injectability attributes. The main conclusion of the study was that some products present qualitatively better injectability attributes than others (i.e., smoother injection curves) and that in the hands of different injectors, the same product may behave very differently in terms of injectability. Overall, the presented work underscored the central importance for injectors to work with HA-based dermal filler products with which they are experienced, comfortable, and skilled, eventually aiming toward enhancing the clinical results and overall patient experience.

## 4. Materials and Methods

### 4.1. Materials Used in the Study

Physiological saline solution (NaCl 0.9%) was purchased from Bichsel (Unterseen, Switzerland). Lidocaine was purchased from Sigma-Aldrich (Buchs, Switzerland). A total of 28 different cross-linked HA-based commercial dermal fillers from the JUVÉDERM^®^, Restylane^®^, BELOTERO^®^, TEOSYAL RHA^®^, and STYLAGE^®^ brands were purchased from the respective product manufacturers. The various needles used in the study were taken directly from each corresponding product packaging and comprised 30 G × ½″ needles (0.30 × 13 mm; TSK Laboratories, Tochigi-Ken, Japan), 29 G × ½″ needles (0.33 × 12 mm; Terumo, Tokyo, Japan), and 27 G × ½″ needles (0.40 × 13 mm; TSK Laboratories, Tochigi-Ken, Japan). For establishing ex vivo injectability measurement conditions, human skin tissue from resected abdominoplasty waste materials was used. For establishing standardized in vitro injectability measurement conditions, SimSkin^®^ synthetic skin was purchased from Wallcur (San Diego, CA, USA). SimSkin^®^ synthetic skin consists of an epidermis, dermis, and subcutaneous layer. Its total thickness is 0.6 mm (i.e., 0.3 mm for the epidermis, 0.2 mm for the dermis, and 0.1 mm for the subcutaneous layer. The exact polymeric composition of the three layers of the SimSkin^®^ product is not provided by the manufacturer.

### 4.2. Comparative Manual Injectability Studies in Ex Vivo Human Skin and in SimSkin^®^ Cutaneous Equivalents

In order to first validate the SimSkin^®^ cutaneous equivalent model for further experimental setup standardization, comparative manual injectability studies were performed in vitro and ex vivo. Therefore, quantitative injectability measurements were performed by two specialized and experienced clinicians using a dynamometric sensor (FlexiForce^®^ Quickstart Board, Tekscan, Boston, MA, USA) connected to myDAQ for data acquisition (National Instruments, Austin, TX, USA). The force injection parameters of a commercially available dermal filler product (i.e., TEOSYAL RHA^®^ 2) were determined using the original syringes and needles used for clinical product administration. The TEOSYAL RHA^®^ 2 product (i.e., based on the “Preserved Network^®^” cross-linking technology; Teoxane, Geneva, Switzerland) was injected into ex vivo human skin samples and in the SimSkin^®^ scaffold. The wrinkle-filling hydrogel (i.e., TEOSYAL RHA^®^ 2) was injected point-by-point into the superficial to medium dermis in a retro-tracing manner. For each injection, the needle was introduced tangentially to the skin plane at an angle < 10°. The mean forces of injection and peak forces of injection were automatically recorded.

### 4.3. Comparative Product Benchmarking: Multi-Injector Manual Injectability Study in Cutaneous Equivalents

A standardized comparative injectability study was performed for the 28 included commercial hydrogel products. The hydrogels were injected into SimSkin^®^ scaffolds by three specialized and experienced clinicians in clinical conditions (i.e., products were injected as if the recipient was a live patient). All products were administered using the original syringes and needles used for clinical product administration. Injectability measurements were performed using a dynamometric sensor (FlexiForce^®^ Quickstart Board; Tekscan, Boston, MA, USA) connected to myDAQ for data acquisition (National Instruments, Austin, TX, USA). The force injection parameters of the commercially available dermal filler products were determined and compared. Injectability results were compared between the various products and between the three injectors.

### 4.4. Comparative Automated Injectability Study in Cutaneous Equivalents at Constant Injection Speed

In order to enhance the setup standardization and the experimental result granularity levels, automated product injectability measurements were performed at constant speed on a Texture Analyzer TA.XT. Plus instrument (Tracomme, Schlieren, Switzerland). The 28 considered hydrogel products were sequentially injected into SimSkin^®^ scaffolds at a constant plunger rod actuation speed of 1 mm·s^−1^ at ambient temperature (i.e., 25 °C). The relatively fast injection speed of 1 mm·s^−1^ was retained from preliminary experiments in order to best discriminate products in terms of hydrogel system intra-syringe homogeneity. The force injection profiles of the various products were determined using the respective original syringes and needles used for clinical product administration.

### 4.5. Comparative Analysis of Different Manual Hydrogel Injection Techniques

In order to complement the automated injectability study results, manual injections were performed by two specialized and experienced clinicians using various injection techniques. Specifically, quantitative injectability studies were performed using a dynamometric sensor (FlexiForce^®^ Quickstart Board; Tekscan, Boston, MA, USA) connected to myDAQ for data acquisition (National Instruments, Austin, TX, USA) and SimSkin^®^ scaffolds. The 28 retained commercial hydrogel products were injected using retro-tracing injections in the dermis and hypodermis and using the bolus technique in the hypodermis. Qualitative assessments of hydrogel product injectability were recorded for each product, injector, and injection technique, focusing on potential variations in the required pressure to inject the product.

### 4.6. Experimental Assessment of the Impact of Lidocaine on Hydrogel System Attributes during Product Sterilization

In order to experimentally assess the impact of lidocaine on hydrogel rheological attributes within the sterilization process, formulations with lidocaine and without lidocaine were prepared. A BDDE-cross-linked HA-based hydrogel (University of Geneva, Geneva, Switzerland) served as an experimental base hydrogel. Lidocaine was incorporated at 2 mg/mL in a fraction of the hydrogel base to approximate the quantity generally present in commercial dermal filler products. Both hydrogel groups were conditioned in 6R clear glass vials and were submitted to steam sterilization (Systec, Sysmex, Kobe, Japan) at 121 °C for 12 min, using rapid ramp heating and cooling protocols. Pre-sterilization and post-sterilization rheological attributes (i.e., storage modulus G′ and loss modulus G″) were determined in oscillatory rheology on a Haake Mars rheometer (Thermo Fisher Scientific, Waltham, MA, USA). A Peltier cone plate characterized by a C35 2°/Ti measuring geometry was mounted on the instrument. The measurements were performed in triplicate at 22 °C on 450 μL of sample with a constant oscillatory frequency of 1 Hz. Shear stress was set at 3 N/m^2^ in order to remain in the linear viscoelastic region. A sample hood was used during the measurements to minimize sample evaporation.

### 4.7. Statistical Analysis and Data Presentation

Data were reported as mean values accompanied by the corresponding standard deviations as error bars. For the statistical comparison of values from multi-group quantitative datasets, a one-way ANOVA or a two-way ANOVA test was performed and was followed by a post hoc Tukey’s multiple comparison test. A *p*-value < 0.05 was retained as a general base for statistical significance determination. Detailed levels of statistical significance can be found in the Results section and in the Supplementary Tables. The statistical calculations and/or data presentation were performed using Microsoft Excel (Microsoft Corporation, Redmond, WA, USA), Microsoft PowerPoint, and GraphPad Prism v. 8.0.2 (GraphPad Software, San Diego, CA, USA).

## Figures and Tables

**Table 1:** Listing of the five manufacturers from which the 28 cross-linked HA-based commercial dermal filler products were obtained. The study focused on well-established brands from highly reputable laboratories with long-term clinical track records and global product commercialization history. CE, European mark of conformity; FDA, US Food and Drug Administration; HA, hyaluronic acid; USA, United States of America.

Parameters	Cross-Linked HA-Based Dermal Filler Product Manufacturers ^1^				
Company Name	Allergan Aesthetics (Subsidiary of AbbVie Inc.)	Galderma SA	Merz Aesthetics (Subsidiary of Merz Group)	Teoxane SA	Laboratoires VIVACY
Company Headquarters	Irvine, CA, USA	Zug, Switzerland	Frankfurt am Main, Germany	Geneva, Switzerland	Paris, France
Product Brand of Interest	JUVÉDERM^®^	Restylane^®^	BELOTERO^®^	TEOSYAL RHA^®^	STYLAGE^®^
Product Brand Launch Year	2000	1996	2005	2004	2008
Product Types	Class III medical device	Class III medical device	Class III medical device	Class III medical device	Class III medical device
Product Approvals	CE-marked; FDA-approved	CE-marked; FDA-approved	CE-marked; FDA-approved	CE-marked; FDA-approved	CE-marked

^1^ Laboratories specified as product legal manufacturers.

**Table 2:** Comparative overview of the technical attributes and characteristics of the cross-linked HA-based commercial dermal filler products included in the study. Within each product brand, formulation-based and clinical indication specificities were found to differentiate the retained injectable products. G, gauge; HA, hyaluronic acid.

Product Brand and Name ^1^	Specified Product Clinical Uses	Needle Gauge (G) ^2^	HA Concentration ^3^	Cross-Linked HA	Contains Lidocaine	Cross-Linking Technology ^4^
JUVÉDERM^®^ VOLBELLA^®^	Fine lines; tear through	30 G	15 mg/mL	Yes	Yes	VYCROSS^®^
JUVÉDERM^®^ VOLIFT^®^	Medium fold; lips	30 G	17.5 mg/mL	Yes	Yes	VYCROSS^®^
JUVÉDERM^®^ VOLUMA^®^	Volumizer	27 G	20 mg/mL	Yes	Yes	VYCROSS^®^
JUVÉDERM^®^ VOLUX^®^	Cheeks; temples; jaw line; chin volumizer	27 G	25 mg/mL	Yes	Yes	VYCROSS^®^
JUVÉDERM^®^ Ultra 2	Medium lines; lip border	30 G	24 mg/mL	Yes	Yes	HYLACROSS^®^
JUVÉDERM^®^ Ultra 3	Deep folds; lip volume	27 G	24 mg/mL	Yes	Yes	HYLACROSS^®^
Restylane^®^	Medium lines	30 G	20 mg/mL	Yes	No	NASHA^®^
Restylane^®^	Medium lines	29 G	20 mg/mL	Yes	No	NASHA^®^
Restylane^®^ Lido	Medium lines	30 G	20 mg/mL	Yes	Yes	NASHA^®^
Restylane^®^ Lido	Medium lines	29 G	20 mg/mL	Yes	Yes	NASHA^®^
Restylane^®^ Lyft	Deep folds; volumizer	27 G	20 mg/mL	Yes	No	NASHA^®^
Restylane^®^ Lyft Lido	Deep folds; volumizer	27 G	20 mg/mL	Yes	Yes	NASHA^®^
BELOTERO^®^ Soft	Fine lines	30 G	20 mg/mL	Yes	No	CPM^®^
BELOTERO^®^ Soft +	Fine lines	30 G	20 mg/mL	Yes	Yes	CPM^®^
BELOTERO^®^ Balance	Medium lines; lip border	30 G	22.5 mg/mL	Yes	No	CPM^®^
BELOTERO^®^ Balance +	Medium lines; lip border	30 G	22.5 mg/mL	Yes	Yes	CPM^®^
BELOTERO^®^ Intense	Deep folds; lip volumizer	27 G	25.5 mg/mL	Yes	No	CPM^®^
BELOTERO^®^ Intense +	Deep folds; lip volumizer	27 G	25.5 mg/mL	Yes	Yes	CPM^®^
BELOTERO^®^ Volume	Volumizer	30 G	26 mg/mL	Yes	No	CPM^®^
BELOTERO^®^ Volume +	Volumizer	30 G	26 mg/mL	Yes	Yes	CPM^®^
TEOSYAL RHA^®^ 1	Fine lines	30 G	15 mg/mL	Yes (mix)	Yes	Preserved Network^®^
TEOSYAL RHA^®^ 2	Medium folds; lip contour	30 G	23 mg/mL	Yes (mix)	Yes	Preserved Network^®^
TEOSYAL RHA^®^ 3	Deep folds; lip volumizer	27 G	23 mg/mL	Yes (mix)	Yes	Preserved Network^®^
TEOSYAL RHA^®^ 4	Volumizer	27 G	23 mg/mL	Yes (mix)	Yes	Preserved Network^®^
TEOSYAL Ultra Deep	Strong volumizer	25 G	25 mg/g	Yes	Yes	Teosyal PureSense
STYLAGE^®^ S	Fine lines	30 G	16 mg/g	Yes	Yes	IPN-Like^®^ + mannitol
STYLAGE^®^ M	Medium folds; lip contour	30 G	20 mg/g	Yes	Yes	IPN-Like^®^ + mannitol
STYLAGE^®^ L	Deep folds; lip volumizer	27 G	24 mg/g	Yes	Yes	IPN-Like^®^ + mannitol
STYLAGE^®^ XL	Volumizer	27 G	26 mg/g	Yes	Yes	IPN-Like^®^ + mannitol
STYLAGE^®^ XXL	Cheeks; temples; jawline; chin volumizer	27 G	21 mg/g	Yes	No	IPN-Like^®^ + mannitol

^1^ All of the data used for the cross-linked HA-based hydrogel product technical comparison work were compiled from manufacturer-provided information. ^2^ Needles as provided by manufacturers in product secondary packaging or as specified by the manufacturer for a given product. ^3^ It is notable that while the HA contents of the various products are specified, details on manufacturer-specific cross-linking technologies and the degrees of system cross-linking constitute trade secrets. ^4^ Various degrees of polymer cross-linking frequently exist within most product brands.

## Data Availability

The data presented in this study are openly available in the article.

## Supplementary Materials

The following supporting information can be downloaded at https://www.mdpi.com/article/10.3390/gels10020101/s1. Figure S1: Illustration of different syringe handling dispositions for dermal filler injection; Figure S2: Force injection profiles of the BELOTERO^®^ products; Figure S3: Force injection profiles of the Restylane^®^ products; Figure S4: Force injection profiles of the JUVÉDERM^®^ products; Figure S5: Force injection profiles of the TEOSYAL RHA^®^ products; Figure S6: Force injection profiles of the STYLAGE^®^ products; Figure S7: Results of experimental sterilization studies; Table S1: Technical benchmarking data on the investigated products; Table S2: Complementary statistical analysis results; Table S3: Complementary statistical analysis results; Table S4: Qualitative and quantitative injectability records for BELOTERO^®^ products; Table S5: Qualitative and quantitative injectability records for Restylane^®^ products; Table S6: Qualitative and quantitative injectability records for STYLAGE^®^ products; Table S7: Qualitative and quantitative injectability records for TEOSYAL RHA^®^ products; Table S8: Qualitative and quantitative injectability records for JUVÉDERM^®^ products; Table S9: Complementary statistical analysis results; Table S10: Complementary statistical analysis results.

## Abbreviations

BDDE	1,4-butanediol diglycidyl ether
CE	European mark of conformity
Da	Daltons
FDA	US Food and Drug Administration
G′	storage modulus
G″	loss modulus
HA	hyaluronic acid
ISO	International Standards Organization
MD	medical device
MDa	megaDaltons
min	minutes
MW	molecular weight
N	Newtons
NA	non-applicable
ns	non-significant
Pa	Pascals
Pa·s	Pascal seconds
ROS	reactive oxygen species
USA	United States of America
