# Supplementary material for: Clinical Perspectives on the Injectability of Cross-Linked Hyaluronic Acid Dermal Fillers: A Standardized Methodology for Commercial Product Benchmarking with Inter-Injector Assessments
Source: Gels. 2024 Jan 26;10(2):101. doi: 10.3390/gels10020101 (PMC10888303; doi:10.3390/gels10020101)
Supplement: Supplementary file 1 [file gels-10-00101-s001.zip › Suppl Mat/Supplementary Materials.pdf]

Supplementary Materials: **Clinical Perspectives on the Injectability of Cross-Linked Hyaluronic Acid Dermal Fillers: A Standardized Methodology for Commercial Product Benchmarking with Inter-Injector Assessments**

Patrick Micheels, Alexandre Porcello, Thierry Bezzola, Daniel Perrenoud, Pierre Quinodoz, Yogeshvar Kalia, Eric Allémann, Alexis Laurent and Olivier Jordan

1. Supplementary Figures

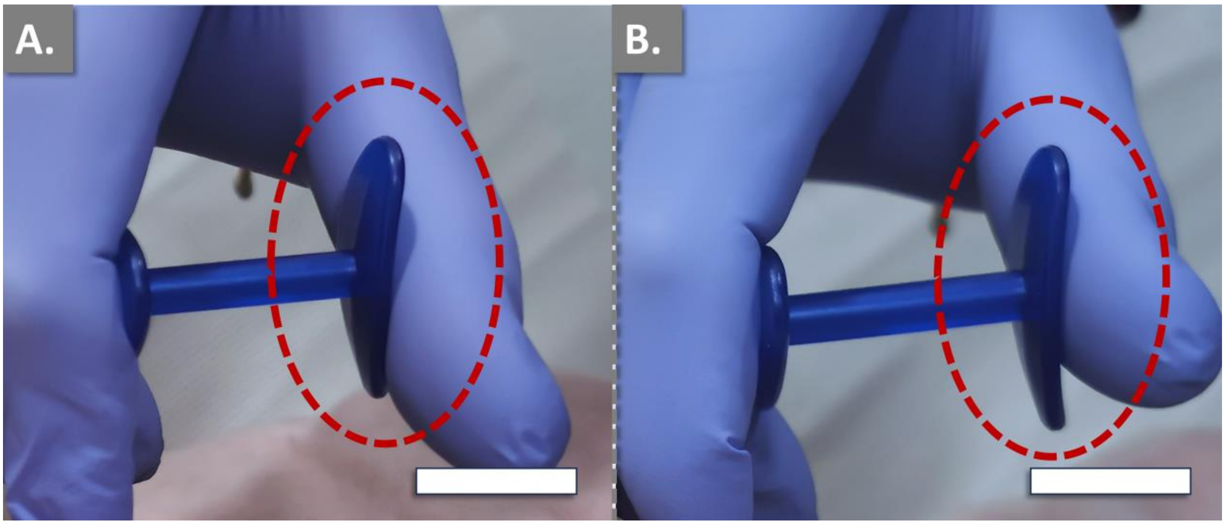

Figure S1. Illustration of different injector thumb positioning options on the plunger rod hilt of the syringe. (**A**) Depiction of an injection position using the P1-P2 joint of the thumb. (**B**) Depiction of an injection position using the pad or pulp of the thumb. Scale bars = 1.5 cm. Reproduced with permission, Patrick Micheels, private archives.

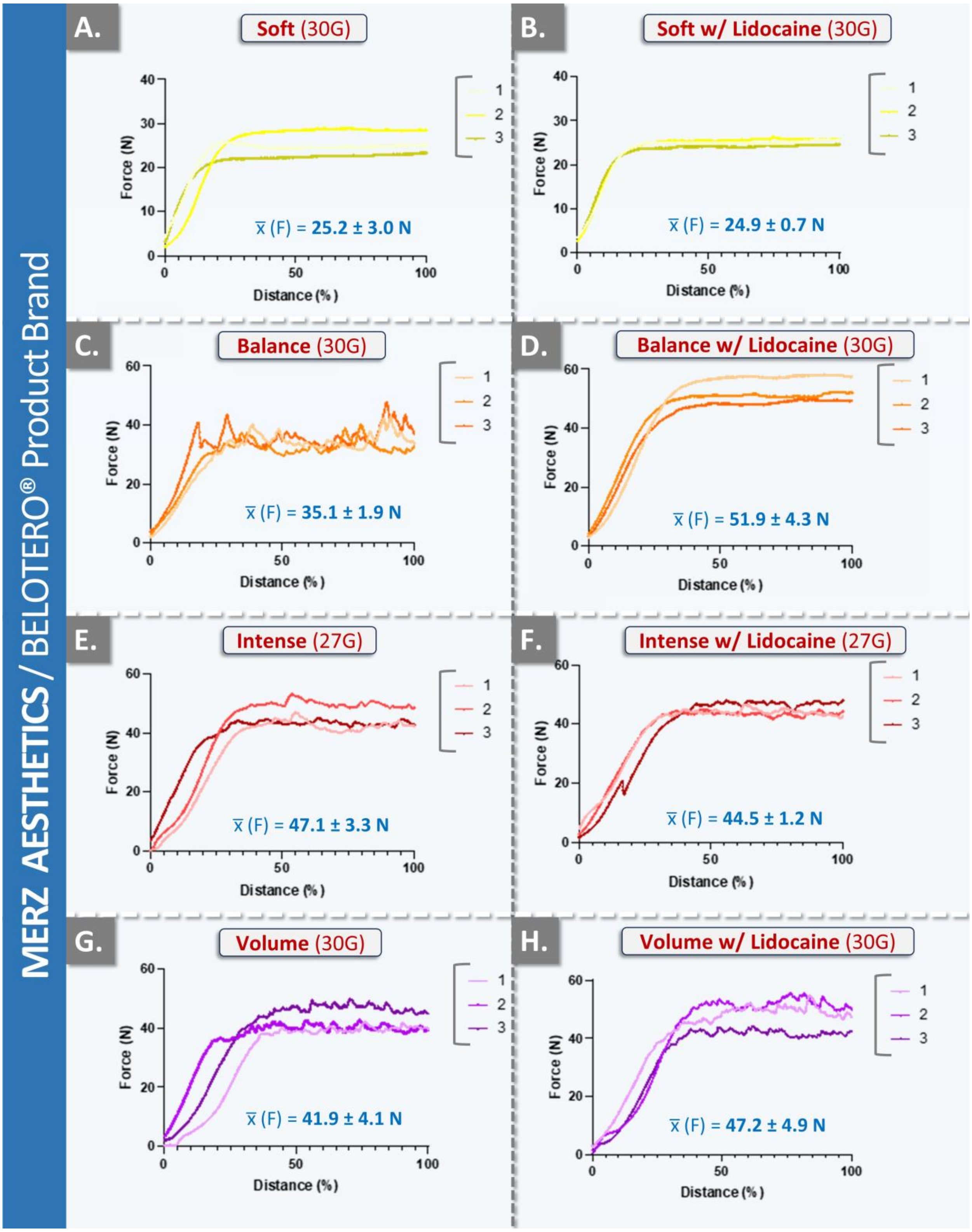

Figure S2. Force injection profiles of various Merz Aesthetics BELOTERO® products in an automated measurement setup, at a constant plunger rod actuation speed of 1 mm·s^{-1}, which was conducted at room temperature. All measurements were performed in triplicate. Force injection profiles of the BELOTERO® Soft product (**A**), the BELOTERO® Soft product with lidocaine (**B**), the BELOTERO® Balance product (**C**), the BELOTERO® Balance product with lidocaine (**D**), the BELOTERO® Intense product (**E**), the BELOTERO® Intense product with lidocaine (**F**), the BELOTERO® Volume product (**G**), and the BELOTERO® Volume product with lidocaine (**H**). F, plateau force; G, gauge; N, Newtons.

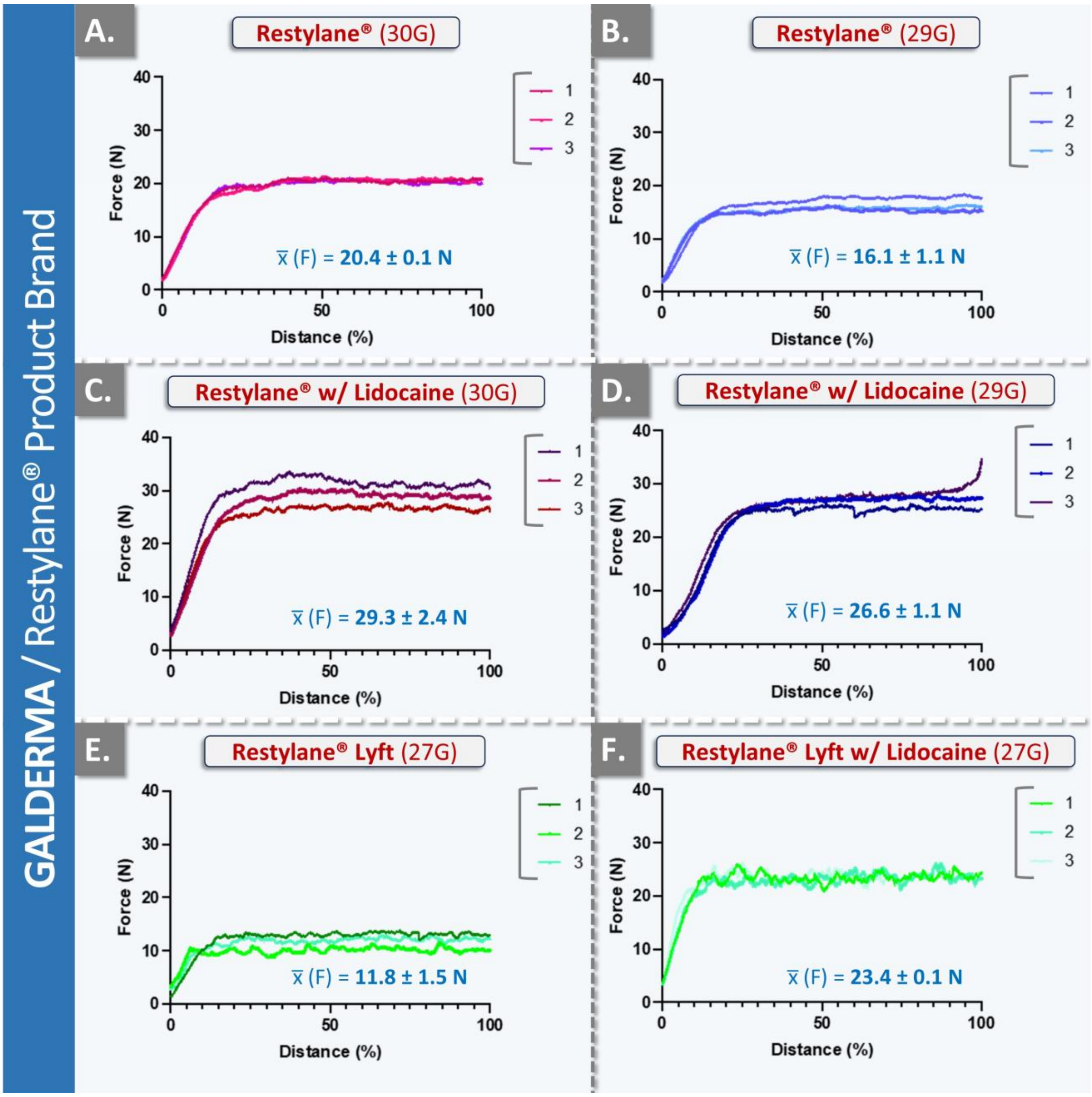

Figure S3. Force injection profiles of various Galderma Restylane® products in an automated measurement setup, at a constant plunger rod actuation speed of 1 mm·s^{-1}, which was conducted at room temperature. All measurements were performed in triplicate. Force injection profiles of the Restylane® product (**A**,**B**), the Restylane® product with lidocaine (**C**,**D**), the Restylane® Lyft product (**E**), and the Restylane® Lyft product with lidocaine (**F**). F, plateau force; G, gauge; N, Newtons.

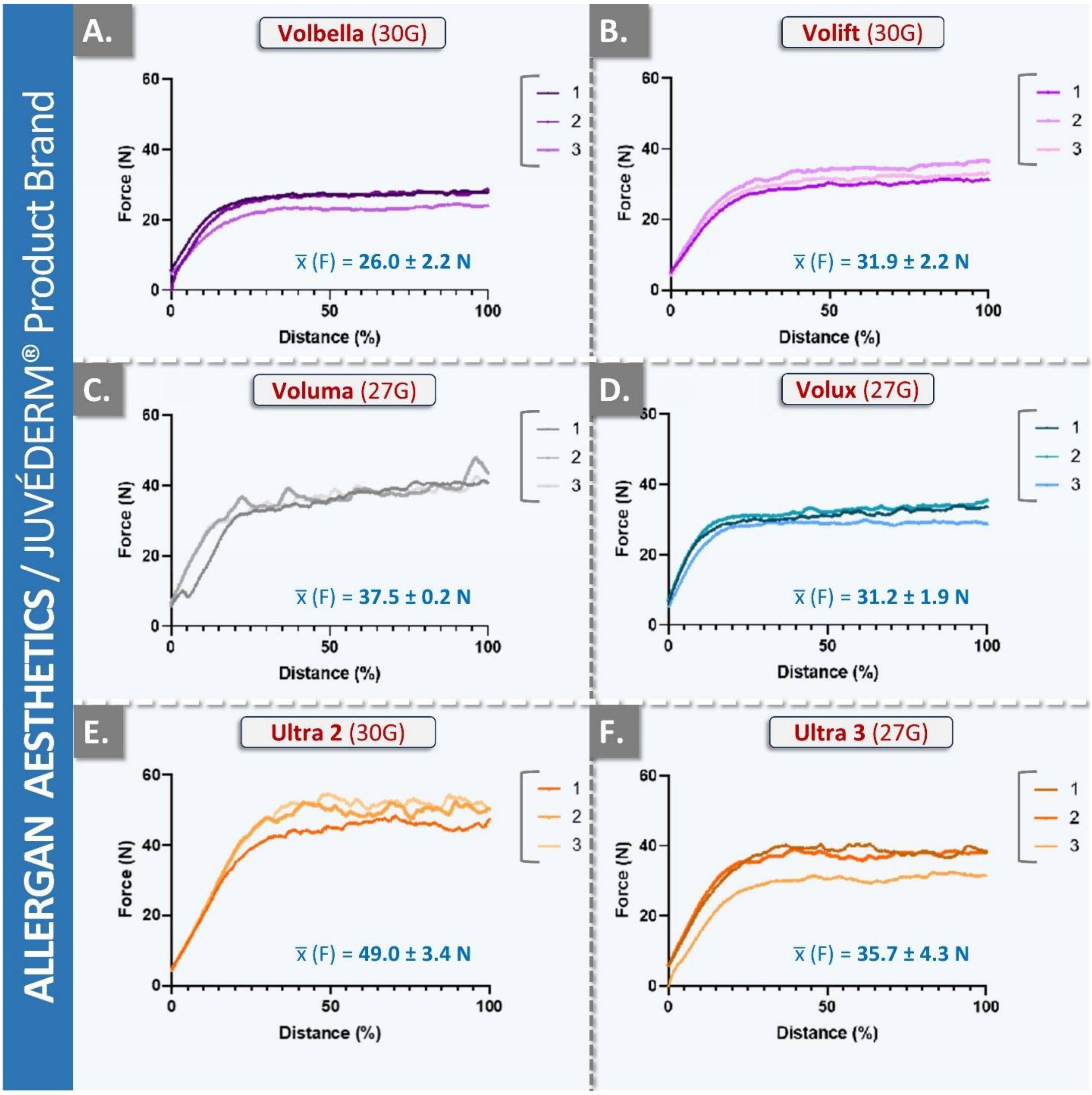

Figure S4. Force injection profiles of various Allergan Aesthetics JUVÉDERM® products in an automated measurement setup, at a constant plunger rod actuation speed of 1 mm·s^{-1}, which was conducted at room temperature. All measurements were performed in triplicate. Force injection profiles of the JUVÉDERM® Volbella product (**A**), the JUVÉDERM® Volift product (**B**), the JUVÉDERM® Voluma product (**C**), the JUVÉDERM® Volux product (**D**), the JUVÉDERM® Ultra 2 product (**E**), and the JUVÉDERM® Ultra 3 product. F, plateau force; G, gauge; N, Newtons.

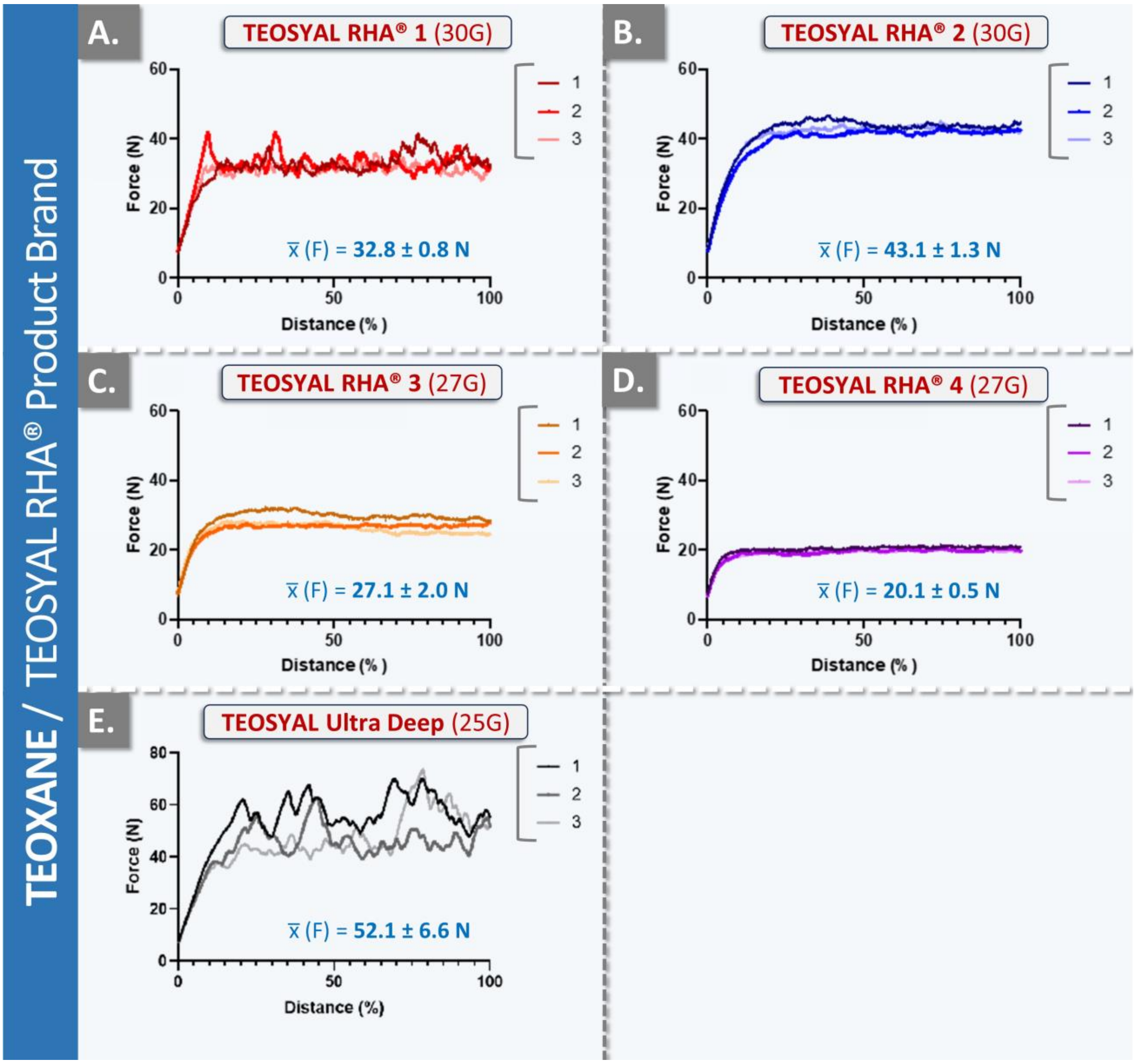

Figure S5. Force injection profiles of various TEOXANE TEOSYAL RHA® products in an automated measurement setup, at a constant plunger rod actuation speed of 1 mm·s^{-1}, which was conducted at room temperature. All measurements were performed in triplicate. Force injection profiles of the TEOSYAL RHA® 1 product (**A**), the TEOSYAL RHA® 2 product (**B**), the TEOSYAL RHA® 3 product (**C**), the TEOSYAL RHA® 4 product (**D**), and the TEOSYAL Ultra Deep product (**E**). F, plateau force; G, gauge; N, Newtons.

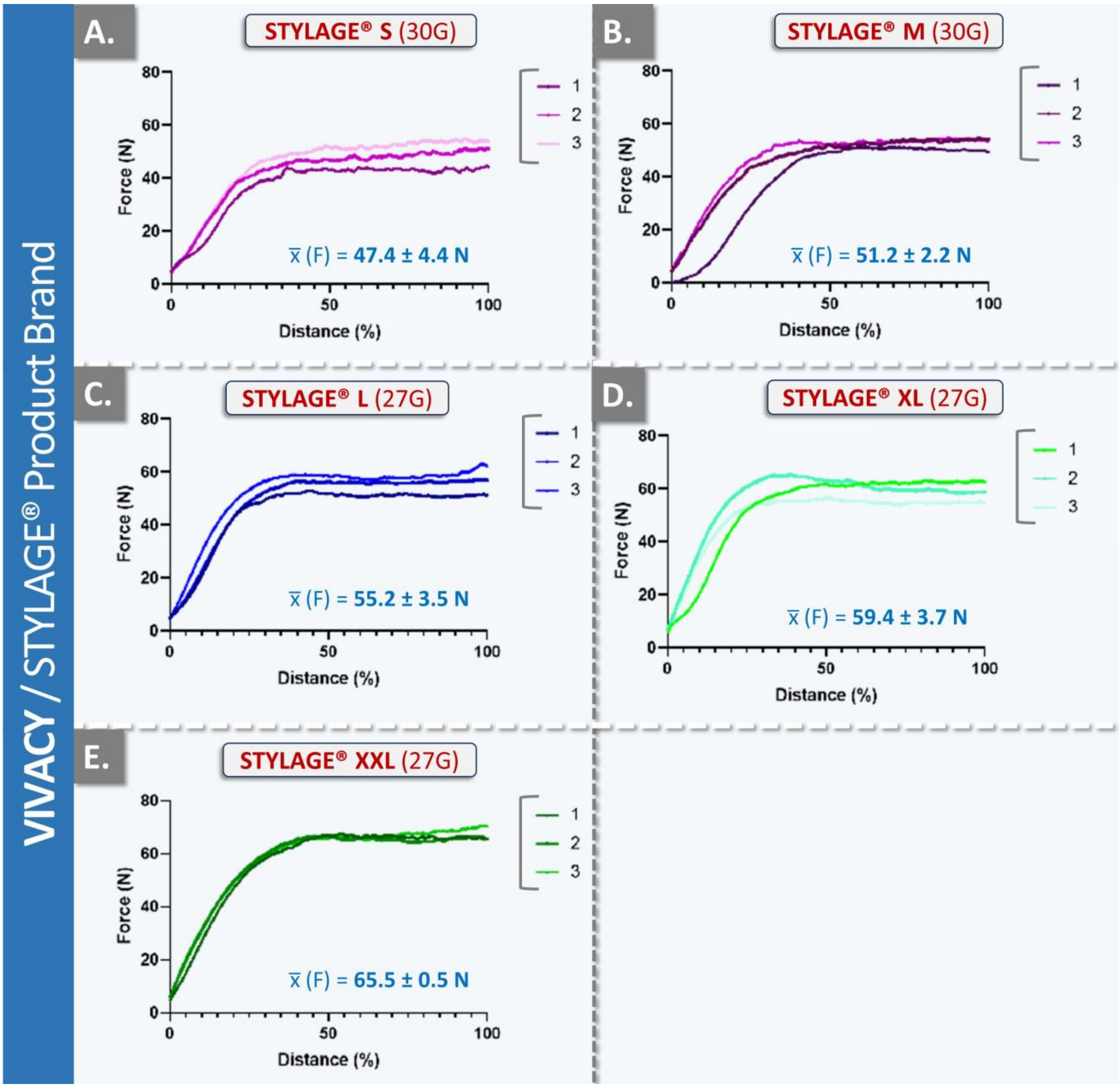

Figure S6. Force injection profiles of various Vivacy STYLAGE® products in an automated measurement setup, at a constant plunger rod actuation speed of 1 mm·s^{-1}, which was conducted at room temperature. All measurements were performed in triplicate. Force injection profiles of the STYLAGE® S product (**A**), the STYLAGE® M product (**B**), the STYLAGE® L product (**C**), the STYLAGE® XL product (**D**), and the STYLAGE® XXL product (**E**). F, plateau force; G, gauge; N, Newtons.

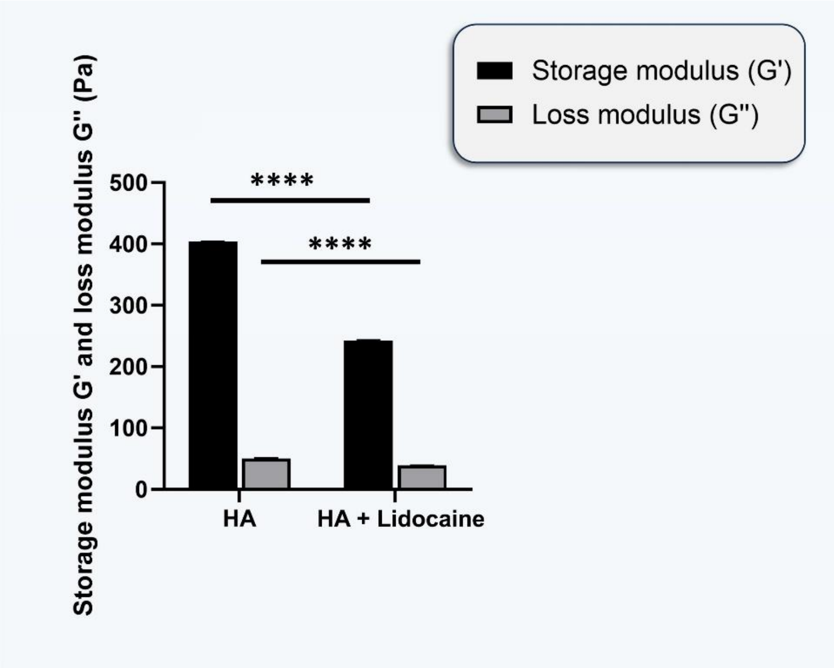

Figure S7. Results of experimental sterilization studies, for the assessment of the impact of lidocaine incorporation in cross-linked HA-based hydrogel systems. Post-sterilization rheological attributes of the hydrogel samples were determined and revealed significantly lower storage and loss moduli values in the samples containing lidocaine. Detailed results of the statistical analyses are presented in Table S10. HA, hyaluronic acid; Pa, Pascals.

2. Supplementary Tables

Table S1. Technical benchmarking of the investigated commercial dermal filler products in terms of physical, chemical, and rheological attributes. From the physical standpoint, all of the considered hydrogels are continuous systems with respective viscous and elastic components. From a chemical standpoint, the considered hydrogels are composed of covalently cross-linked polymeric networks, hydrated with injection-grade, osmotically controlled, and pH-controlled aqueous media. In case of presence of small molecule additives (e.g., mannitol, lidocaine), these are dissolved in the continuous phase of the hydrogel and may interact with the polymeric network via multiple chemical bonds and mechanisms (i.e., protective for mannitol, pro-degradant under specific conditions for lidocaine). BDDE, 1,4-butanediol diglycidyl ether; HA, hyaluronic acid; NA, non-applicable; RT, room temperature.

Product Brand and Name	Cross-Linking Agent	Storage Modulus G′ (Pa)	Loss Modulus G″ (Pa)	Tan δ	Frequency (Hz)	Temperature (°C)	References
JUVÉDERM® VOLBELLA®	BDDE	271	39	0.144	5	RT	[43]
JUVÉDERM® VOLIFT®	BDDE	340	46	0.135	5	RT	[43]
JUVÉDERM® VOLUMA®	BDDE	354	38	0.109	1	37	[58]
JUVÉDERM® VOLUX®	BDDE	665	49	0.074	5	RT	[43]
JUVÉDERM® Ultra 2	BDDE	188	75	0.399	5	RT	[43]
JUVÉDERM® Ultra 3	BDDE	173	37	0.210	0.1	37	[42]
Restylane®	BDDE	864	185	0.214	5	RT	[43]
Restylane® Lido	BDDE	NA	NA	NA	NA	NA	NA
Restylane® Lyft	BDDE	977	198	0.203	5	RT	[43]
Restylane® Lyft Lido	BDDE	NA	NA	NA	NA	NA	NA
BELOTERO® Soft	BDDE	7	9	1.270	0.1	37	[42]
BELOTERO® Soft +	BDDE	40	42	1.050	5	RT	[43]
BELOTERO® Balance	BDDE	81	64	0.790	4.6	25	[58]
BELOTERO® Balance +	BDDE	89	63	0.705	4.6	25	[58]
BELOTERO® Intense	BDDE	76	27	0.350	0.1	37	[42]
BELOTERO® Intense +	BDDE	255	110	0.431	5	RT	[43]
BELOTERO® Volume	BDDE	233	54	0.230	0.7	37	[43]
BELOTERO® Volume +	BDDE	438	103	0.235	5	NA	[43]
TEOSYAL RHA® 1	BDDE	133	54	0.406	5	RT	[43]
TEOSYAL RHA® 2	BDDE	319	99	0.310	5	RT	[43]
TEOSYAL RHA® 3	BDDE	264	67	0.254	5	RT	[43]
TEOSYAL RHA® 4	BDDE	346	62	0.179	5	RT	[43]
TEOSYAL Ultra Deep	BDDE	348	54	0.155	5	RT	[43]
STYLAGE® S	BDDE	165	31	0.190	1	25	[46]
STYLAGE® M	BDDE	195	35	0.180	1	25	[46]
STYLAGE® L	BDDE	240	41	0.170	1	25	[46]
STYLAGE® XL	BDDE	290	46	0.160	1	25	[46]
STYLAGE® XXL	BDDE	290	38	0.130	1	25	[46]

Table S2. Quantitative results of the post hoc Tukey's multiple comparison test, in relation with the comparative injectability setup study (i.e., ex vivo human skin versus synthetic SimSkin® substrates) presented in Figure 1. Non-significant differences (i.e., corresponding to a p-value > 0.05) were not listed. N, Newtons.

**Compared Parameters**	**Compared Groups / Injectors**	**Mean Absolute Difference** (N)	**Adjusted *p*-Value**	**Statistical Significance Level** [1]
Mean Force	Ex vivo – Injector 2 vs. SimSkin® – Injector 2	0.2767	0.0330	*
	SimSkin® – Injector 1 vs. SimSkin® – Injector 2	0.2833	0.0294	*
Peak Force	Ex vivo – Injector 2 vs. SimSkin® – Injector 2	0.5667	0.0462	*

[1] A statistical significance level described by one asterisk "*" corresponded to a p-value between 0.01 and 0.05.

Table S3. Quantitative results of the post hoc Tukey's multiple comparison test, in relation with the comparative manual injectability data presented in Figure 2 and Figure 3. Each dermal filler product was statistically compared to all the other products of the same brand. Non-significant differences (i.e., corresponding to a *p*-value > 0.05) were not listed. DP, Daniel Perrenoud; G, gauge; L, lidocaine; PM, Patrick Micheels; TB, Thierry Bezzola.

Injectors	Product Brands	Compared Products	Mean Absolute Difference (N)	Adjusted *p*-Value	Statistical Significance Level [1]
PM	JUVÉDERM®	VOLBELLA® vs. VOLIFT®	−8.599	< 0.0001	****
		VOLBELLA® vs. Ultra 3	−3.400	0.0393	*
		VOLIFT® vs. VOLUMA®	8.288	< 0.0001	****
		VOLIFT® vs. VOLUX®	6.262	0.0004	***
		VOLIFT® vs. Ultra 3	5.199	0.0018	**
		VOLIFT® vs. Ultra 2	8.288	< 0.0001	****
	Restylane®	29G + L vs. 30G	−1.658	0.0002	***
		29G vs. 30G + L	−1.403	0.0011	**
		29G vs. 30G	−2.253	< 0.0001	****
		30G + L vs. 30G	−0.850	0.0425	*
		30G + L vs. Lyft 27G	1.289	0.0022	**
		30G vs. Lyft 27G + L	1.530	0.0005	***
		30G vs. Lyft 27G	2.139	< 0.0001	****
	BELOTERO®	Soft + L vs. Balance + L	−10.640	0.0127	*
		Soft vs. Balance + L	−15.560	0.0024	**
		Soft vs. Balance	−10.640	0.0127	*
		Balance + L vs. Intense	12.200	0.0219	*
		Balance + L vs. Volume + L	11.100	0.0088	**
		Balance + L vs. Volume	16.020	0.0017	**
		Balance vs. Volume	11.100	0.0088	**
	TEOSYAL®	RHA® 1 vs. RHA® 3	−1.955	0.0031	**
		RHA® 3 vs. RHA® 4	1.473	0.0164	*
	STYLAGE®	S vs. M	2.451	0.0122	*
		S vs. L	3.542	0.0009	***
		S vs. XXL	2.153	0.0268	*
		L vs. XL	−2.253	0.0206	*
TB	JUVÉDERM®	VOLIFT® vs. VOLUX®	7.834	< 0.0001	****
		VOLIFT® vs. VOLBELLA®	7.296	< 0.0001	****
		VOLIFT® vs. VOLUMA®	9.095	< 0.0001	****
		VOLIFT® vs. Ultra 2	4.236	0.0016	**
		VOLUX® vs. Ultra 3	−9.152	< 0.0001	****
		VOLUX® vs. Ultra 2	−3.598	0.0059	**
		VOLBELLA® vs. Ultra 3	−8.613	< 0.0001	****
		VOLBELLA® vs. Ultra 2	−3.060	0.0189	*
		VOLUMA® vs. Ultra 3	−10.410	< 0.0001	****
		VOLUMA® vs. Ultra 2	−4.859	0.0005	***
		Ultra 3 vs. Ultra 2	5.553	0.0001	***
	Restylane®	29G + L vs. 29G	2.904	0.0104	*
		29G + L vs. Lyft 27G + L	4.080	0.0007	***
		29G + L vs. Lyft 27G	4.009	0.0008	***
		29G vs. 30G + L	−2.408	0.0359	*
		29G vs. 30G	−2.692	0.0177	*
		30G + L vs. Lyft 27G + L	3.584	0.0020	**
		30G + L vs. Lyft 27G	3.513	0.0024	**
		30G vs. Lyft 27G + L	3.868	0.0011	**

		30G vs. Lyft 27G	3.797	0.0012	**
	BELOTERO®	Soft + L vs. Balance + L	–5.667	< 0.0001	****
		Soft + L vs. Intense + L	–3.046	0.0050	**
		Soft + L vs. Intense	–8.004	< 0.0001	****
		Soft + L vs. Volume + L	–5.341	< 0.0001	****
		Soft vs. Balance + L	–6.644	< 0.0001	****
		Soft vs. Balance	–3.060	0.0048	**
		Soft vs. Intense + L	–4.023	0.0003	***
		Soft vs. Intense	–8.982	< 0.0001	****
		Soft vs. Volume + L	–6.318	< 0.0001	****
		Soft vs. Volume	–2.267	0.0497	*
		Balance + L vs. Balance	3.584	0.0010	**
		Balance + L vs. Intense + L	2.621	0.0177	*
		Balance + L vs. Intense	–2.338	0.0405	*
		Balance + L vs. Volume	4.378	0.0001	***
		Balance vs. Intense	–5.922	< 0.0001	****
		Balance vs. Volume + L	–3.258	0.0027	**
		Intense + L vs. Intense	–4.958	< 0.0001	****
		Intense + L vs. Volume + L	–2.295	0.0458	*
		Intense vs. Volume + L	2.663	0.0156	*
		Intense vs. Volume	6.715	< 0.0001	****
		Volume + L vs. Volume	4.052	0.0003	***
	TEOSYAL®	RHA® 1 vs. RHA® 3	2.947	0.0033	**
		RHA® 1 vs. RHA® 4	4.293	0.0003	***
		RHA® 2 vs. RHA® 3	2.848	0.0041	**
		RHA® 2 vs. RHA® 4	4.193	0.0003	***
	STYLAGE®	S vs. M	–7.834	< 0.0001	****
		S vs. XL	–3.457	0.0176	*
		M vs. L	7.423	< 0.0001	****
		M vs. XL	4.378	0.0037	**
		M vs. XXL	6.063	0.0003	***
		L vs. XL	–3.046	0.0365	*
DP	JUVÉDERM®	VOLUX® vs. VOLUMA®	–4.023	0.0149	*
		VOLBELLA® vs. VOLUMA®	–6.035	0.0006	***
		VOLUMA® vs. Ultra 3	6.007	0.0006	***
		VOLUMA® vs. Ultra 2	5.270	0.0019	**
	BELOTERO®	Soft + L vs. Soft	–7.820	0.0004	***
		Soft + L vs. Balance	–7.069	0.0012	**
		Soft + L vs. Intense + L	–9.534	< 0.0001	****
		Soft + L vs. Volume + L	–11.150	< 0.0001	****
		Soft + L vs. Volume	–4.675	0.0417	*
		Soft vs. Balance + L	6.191	0.0045	**
		Soft vs. Intense	7.551	0.0006	***
		Balance + L vs. Balance	–5.440	0.0136	*
		Balance + L vs. Intense + L	–7.905	0.0004	***
		Balance + L vs. Volume + L	–9.520	< 0.0001	****
		Balance vs. Intense	6.800	0.0018	**
		Intense + L vs. Intense	9.265	< 0.0001	****
		Intense + L vs. Volume	4.859	0.0319	*
		Intense vs. Volume + L	–10.880	< 0.0001	****
		Volume + L vs. Volume	6.474	0.0029	**
	TEOSYAL®	RHA® 1 vs. RHA® 3	4.604	0.0090	**
		RHA® 2 vs. RHA® 3	5.270	0.0040	**

		RHA® 3 vs. RHA® 4	−5.766	0.0023	**
	STYLAGE®	M vs. L	6.928	0.0008	***
		L vs. XL	−3.783	0.0442	*
		L vs. XXL	−6.828	0.0009	***

[1] A statistical significance level described by one asterisk "*" corresponded to a *p*-value between 0.01 and 0.05. A statistical significance level described by two asterisks "**" corresponded to a *p*-value between 0.001 and 0.01. A statistical significance level described by three asterisks "***" corresponded to a *p*-value between 0.0001 and 0.001. A statistical significance level described by four asterisks "****" corresponded to a *p*-value inferior to 0.0001.

Table S4. Comparative analysis of manual injection pressures (i.e., two independent injectors) across different Merz Aesthetics BELOTERO® dermal filler products and injection techniques. Experiments were performed under clinical conditions, using SimSkin® cutaneous equivalents. PM, Patrick Micheels; TB, Thierry Bezzola.

Products	Injection Location	Injection Technique	Needle Gauge (G)	Clinical Assessments and Recorded Pressure Values (N)
BELOTERO® Soft w/o lidocaine	Dermis	Point-by-point	30G	Low pressures, close to 1.00 N
BELOTERO® Soft w/o lidocaine	Dermis	Retrograde tracing	30G	Low variability in injection curves, low pressures close to 1.00 N
BELOTERO® Soft w/o lidocaine	Hypodermis	Bolus	30G	Low pressures, close to 1.50 N. Variability noted in the injection curves
BELOTERO® Soft w/o lidocaine	Hypodermis	Retrograde tracing	30G	Low pressures, close to 1.50 N. Variability noted in the injection curves
BELOTERO® Soft w/ lidocaine	Dermis	Point-by-point	30G	Pressures are double those obtained with the lidocaine-free variant
BELOTERO® Soft w/ lidocaine	Dermis	Retrograde tracing	30G	Slight variability noted in the injection curves
BELOTERO® Soft w/ lidocaine	Hypodermis	Bolus	30G	Pressures are higher than those obtained with the lidocaine-free variant, close to 3.90 N
BELOTERO® Soft w/ lidocaine	Hypodermis	Retrograde tracing	30G	Pressures are higher than those obtained with the lidocaine-free variant, close to 3.90 N
BELOTERO® Balance w/o lidocaine	Dermis	Point-by-point & Retrograde tracing	30G	Pressures are 2.50 N for point-by-point injection and 1.98 N for retrograde tracing injection (homogeneous injection curves)
BELOTERO® Balance w/o lidocaine	Hypodermis	Bolus	30G	Low variability in injection curves, low pressures close to 1.50 N
BELOTERO® Balance w/o lidocaine	Hypodermis	Retrograde tracing	30G	Pressures are 2.79 N with homogeneous injection curves
BELOTERO® Balance w/ lidocaine	Dermis	Point-by-point	30G	Pressures are higher than those obtained with the lidocaine-free variant, close to 4.00 N, with homogeneous injection curves
BELOTERO® Balance w/ lidocaine	Dermis	Retrograde tracing	30G	Pressures are higher than those obtained with the lidocaine-free variant, close to 3.00 N, with inhomogeneous injection curves
BELOTERO® Balance w/ lidocaine	Hypodermis	Bolus	30G	Pressures are higher than those obtained with the lidocaine-free variant, close to 4.00 N, with inhomogeneous injection curves
BELOTERO® Balance w/ lidocaine	Hypodermis	Retrograde tracing	30G	Pressures are higher than those obtained with the lidocaine-free variant, close to 4.00 N, with inhomogeneous injection curves
BELOTERO® Intense w/o lidocaine	Dermis	Point-by-point	27G	Pressures are high, attaining 5.50 N, with inhomogeneous injection curves
BELOTERO® Intense w/o lidocaine	Dermis	Retrograde tracing	27G	Pressures are high, attaining 5.30 N, with inhomogeneous injection curves
BELOTERO® Intense w/o lidocaine	Hypodermis	Bolus	27G	Pressures are high, attaining 6.50 N, with inhomogeneous injection curves

Products	Injection Location	Injection Technique	Needle Gauge (G)	Clinical Assessments and Recorded Pressure Values (N)
BELOTERO® Intense w/o lidocaine	Hypodermis	Retrograde tracing	27G	Pressures are high, attaining 6.50 N, with inhomogeneous injection curves
BELOTERO® Intense w/ lidocaine	Dermis	Point-by-point	27G	Pressures are moderate, attaining 3.60 N, with inhomogeneous injection curves
BELOTERO® Intense w/ lidocaine	Dermis	Retrograde tracing	27G	Lower pressures than the lidocaine-free variant, with homogeneous injection curves
BELOTERO® Intense w/ lidocaine	Hypodermis	Bolus	27G	Lower pressures than the lidocaine-free variant, with inhomogeneous injection curves
BELOTERO® Intense w/ lidocaine	Hypodermis	Retrograde tracing	27G	Inhomogeneous injection curves
BELOTERO® Volume w/o lidocaine	Dermis	Point-by-point	30G	Low pressures, close to 2.00 N
BELOTERO® Volume w/o lidocaine	Dermis	Retrograde tracing	30G	Moderate pressures, close to 2.80 N, with inhomogeneous injection curves
BELOTERO® Volume w/o lidocaine	Hypodermis	Bolus	30G	Moderate pressures, close to 3.00 N, with inhomogeneous injection curves
BELOTERO® Volume w/o lidocaine	Hypodermis	Retrograde tracing	30G	Low pressures, with homogeneous injection curves
BELOTERO® Volume w/ lidocaine	Dermis	Point-by-point	30G	Pressures are comparable to those of the lidocaine-free variant, with inhomogeneous injection curves
BELOTERO® Volume w/ lidocaine	Dermis	Retrograde tracing	30G	Pressures are higher than those of the lidocaine-free variant, with inhomogeneous injection curves
BELOTERO® Volume w/ lidocaine	Hypodermis	Bolus	30G	Pressures are low, with inhomogeneous injection curves
BELOTERO® Volume w/ lidocaine	Hypodermis	Retrograde tracing	30G	Pressures are low, with inhomogeneous injection curves
BELOTERO® Volume w/ lidocaine	Hypodermis	Bolus	27G	Pressures are very low, under 1.00 N, with inhomogeneous injection curves
BELOTERO® Volume w/ lidocaine	Hypodermis	Retrograde tracing	27G	Pressures are very low, under 1.00 N, with inhomogeneous injection curves

Table S5. Comparative analysis of manual injection pressures across different Galderma Restylane® dermal filler products and injection techniques. Experiments were performed under clinical conditions, using SimSkin® cutaneous equivalents. L, lidocaine.

Products	Injection Location	Injection Technique	Needle Gauge (G)	Clinical Assessments and Recorded Pressure Values (N)
Restylane®	Dermis	Point-by-point	30G	Similar pressure (close to 2.00 N)
Restylane®	Dermis	Retrograde tracing	29G	Lower pressure (close to 1.00 N)
Restylane® + L	Dermis	Point-by-point	30G	Higher pressure than w/o lidocaine
Restylane® + L	Dermis	Retrograde tracing	30G	Lower pressure than point-by-point, but higher than w/o lidocaine
Restylane® Lyft	Dermis	Point-by-point	27G	Extremely low pressure, higher than Restylane® w/o lidocaine
Restylane® Lyft	Dermis	Retrograde tracing	27G	Same as point-by-point
Restylane® Lyft + L	Dermis	Point-by-point	27G	Extremely low pressure, slightly higher than w/o lidocaine
Restylane® Lyft + L	Dermis	Retrograde tracing	27G	Same as point-by-point, but less uniform curve than w/o lidocaine
Restylane®	Hypodermis	Bolus & Retrograde tracing	29G	Low pressure (around 1.50 N)
Restylane®	Hypodermis	Bolus & Retrograde tracing	30G	Slightly higher pressure (around 2.90 N)
Restylane® + L	Hypodermis	Bolus & Retrograde tracing	29G	Slightly higher pressure than w/o lidocaine (around 2.97 N)
Restylane® + L	Hypodermis	Bolus & Retrograde tracing	30G	Higher pressure for bolus (around 4.50 N) and less uniform curve than 29 G, but stable and uniform for retrograde tracing (around 2.80 N)
Restylane® Lyft	Hypodermis	Bolus & Retrograde tracing	27G	Extremely low pressure (less than 1.00 N)
Restylane® Lyft + L	Hypodermis	Bolus & Retrograde tracing	27G	Slightly higher pressure than w/o lidocaine and less uniform curve for retrograde tracing

Table S6. Comparative analysis of manual injection pressures (i.e., two independent injectors) across different VIVACY STYLAGE® dermal filler products and injection techniques. Experiments were performed under clinical conditions, using SimSkin® cutaneous equivalents. NA, non-applicable; PM, Patrick Micheels; TB, Thierry Bezzola.

Products	Injection Location	Injection Technique	Needle Gauge (G)	Clinical Assessments and Recorded Pressure Values (N)
STYLAGE® S	Dermis	Point-by-point	30G	3.00 N (Very stable, with minimal variations)
STYLAGE® S	Dermis	Retrograde tracing	30G	3.00 N (Very stable, with very low amplitude variations)
STYLAGE® M	Dermis	Point-by-point	30G	1.80 N
STYLAGE® M	Dermis	Retrograde tracing	30G	3.70 N (Small fluctuations throughout the injection, with low amplitude variations)
STYLAGE® L	Dermis	Point-by-point	27G	2.00 N
STYLAGE® L	Dermis	Retrograde tracing	27G	2.30 N (Very stable, with very low amplitude variations)
STYLAGE® XL	Dermis	Point-by-point	27G	7.00 N
STYLAGE® XL	Dermis	Retrograde tracing	27G	2.80–4.00 N
STYLAGE® XXL	Dermis	Point-by-point	27G	2.80–3.50 N
STYLAGE® XXL	Dermis	Retrograde tracing	27G	2.70 N
STYLAGE® S	Hypodermis	Bolus injection	30G	3.80 N
STYLAGE® S	Hypodermis	Retrograde tracing	30G	Very stable, with very low amplitude variations
STYLAGE® M	Hypodermis	Bolus injection	30G	One injector has ascending curves with very low pressure fluctuations, the other injector is stable from start with very low pressure fluctuations
STYLAGE® M	Hypodermis	Retrograde tracing	30G	Very low pressure fluctuations for one injector, higher for the other with very stable curve
STYLAGE® L	Hypodermis	Bolus injection	27G	Ascending curves, with very low pressure fluctuations
STYLAGE® L	Hypodermis	Retrograde tracing	27G	Slightly ascending curves, with very low amplitude fluctuations
STYLAGE® XL	Hypodermis	Bolus injection	27G	Slightly ascending curves, with some more pronounced pressure fluctuations for both injectors

Table S7. Comparative analysis of manual injection pressures (i.e., two independent injectors) across different TEOXANE TEOSYAL RHA® and Ultra Deep dermal filler products and injection techniques. Experiments were performed under clinical conditions, using SimSkin® cutaneous equivalents. NA, non-applicable; PM, Patrick Micheels; TB, Thierry Bezzola.

Products	Injection Location	Injection Technique	Needle Gauge (G)	Clinical Assessments and Recorded Pressure Values (N)
TEOSYAL RHA® 1	Dermis	Point-by-point	30G	PM: < 1.00 N<br>TB: 3.00 N
TEOSYAL RHA® 1	Dermis	Retrograde tracing	30G	PM: 0.80 N<br>TB: 2.90 N
TEOSYAL RHA® 2	Dermis	Point-by-point	30G	PM: 1.50 N<br>TB: 3.00 N
TEOSYAL RHA® 2	Dermis	Retrograde tracing	30G	1.80 N
TEOSYAL RHA® 3	Dermis	Point-by-point	27G	1.50 N
TEOSYAL RHA® 3	Dermis	Retrograde tracing	27G	PM: 0.58 N<br>TB: 1.50 N
TEOSYAL RHA® 4	Dermis	Point-by-point	27G	PM: < 0.40 N<br>TB: 0.80 N
TEOSYAL RHA® 4	Dermis	Retrograde tracing	27G	PM: < 0.40 N<br>TB: 0.80 N
TEOSYAL RHA® 1	Hypodermis	Bolus	30G	PM: < 1.00 N
TEOSYAL RHA® 1	Hypodermis	Retrograde tracing	30G	PM: Slightly higher than bolus
TEOSYAL RHA® 2	Hypodermis	Bolus	30G	3.40 N
TEOSYAL RHA® 2	Hypodermis	Retrograde tracing	30G	3.40 N
TEOSYAL RHA® 3	Hypodermis	Bolus	27G	1.60 N
TEOSYAL RHA® 3	Hypodermis	Retrograde tracing	27G	1.50 N
TEOSYAL RHA® 4	Hypodermis	Bolus	27G	0.60 N
TEOSYAL RHA® 4	Hypodermis	Retrograde tracing	27G	Very low pressure, very low amplitude variation
TEOSYAL Ultra Deep	Dermis	This gel is not indicated to be injected superficially	25G	NA
TEOSYAL Ultra Deep	Hypodermis	Bolus	25G	1.20 N
TEOSYAL Ultra Deep	Hypodermis	Retrograde tracing	25G	3.00 N

Table S8. Comparative analysis of manual injection pressures (i.e., two independent injectors) across different Allergan Aesthetics JUVÉDERM® dermal filler products and injection techniques. Experiments were performed under clinical conditions, using SimSkin® cutaneous equivalents. PM, Patrick Micheels; TB, Thierry Bezzola.

Product	Injection Location	Injection Technique	Needle Gauge (G)	Clinical Assessments and Recorded Pressure Values (N)
JUVÉDERM® Ultra 2	Dermis	Point-by-point	30G	PM: 0.50 N<br>TB: 2.80 N
JUVÉDERM® Ultra 2	Dermis	Retrograde tracing	30G	PM: 0.55 N<br>TB: 3.50 N
JUVÉDERM® Ultra 3	Dermis	Point-by-point	27G	PM: 2.50 N (1.75–3.60 N)<br>TB: 7.00 N
JUVÉDERM® Ultra 3	Dermis	Retrograde tracing	27G	PM: 3.00 N (beginning) – 1.50 N (end)<br>TB: 7.20 N
JUVÉDERM® Ultra 2	Hypodermis	Bolus	30G	PM: peak at 1.20 N<br>TB: peak at 5.20 N
JUVÉDERM® Ultra 2	Hypodermis	Retrograde tracing	30G	PM: 0.90 N<br>TB: 4.50 N
JUVÉDERM® Ultra 3	Hypodermis	Bolus	27G	PM: peak at 6.85 N<br>TB: peak at 3.61 N
JUVÉDERM® Ultra 3	Hypodermis	Retrograde tracing	27G	PM: 5.58 N<br>TB: decreasing from 3.59 N to 1.80 N
JUVÉDERM® Volbella	Dermis	Retrograde tracing	30G	PM: 0.90 N<br>TB: range of 1.50–1.86 N

Table S9. Quantitative results of the post hoc Tukey's multiple comparison test, in relation with the comparative automated injectability data presented in Figure 4 and Figure 5. Each dermal filler product was statistically compared to all the other products of the same brand. Non-significant differences (i.e., corresponding to a *p*-value > 0.05) were not listed. G, gauge; L, lidocaine.

Product Brands	Compared Products	Mean Absolute Difference (N)	Adjusted *p*-Value	Statistical Significance Level [1]
JUVÉDERM®	VOLIFT® vs. Ultra 2	–17.070	< 0.0001	****
	VOLUX® vs. Ultra 2	–17.770	< 0.0001	****
	VOLBELLA® vs. VOLUMA®	–11.520	0.0022	**
	VOLBELLA® vs. Ultra 3	–9.715	0.0085	**
	VOLBELLA® vs. Ultra 2	–23.030	< 0.0001	****
	VOLUMA® vs. Ultra 2	–11.510	0.0023	**
	Ultra 3 vs. Ultra 2	–13.320	0.0006	***
Restylane®	Lyft vs. Lyft + L	–11.620	< 0.0001	****
	Lyft vs. 29G	–4.294	0.0165	*
	Lyft vs. 29G + L	–17.470	< 0.0001	****
	Lyft vs. 30G	–8.548	< 0.0001	****
	Lyft vs. 30G + L	–14.750	< 0.0001	****
	Lyft + L vs. 29G	7.322	0.0002	***
	Lyft + L vs. 29G + L	–5.855	0.0015	**
	29G vs. 29G + L	–13.180	< 0.0001	****
	29G vs. 30G	–4.254	0.0175	*
	29G vs. 30G + L	–10.450	< 0.0001	****
	29G + L vs. 30G	8.923	< 0.0001	****
	30G vs. 30G + L	–6.198	0.0009	***
BELOTERO®	Soft vs. Balance	–9.882	0.0317	*
	Soft vs. Balance + L	–26.640	< 0.0001	****
	Soft vs. Intense	–21.920	< 0.0001	****
	Soft vs. Intense + L	–19.280	< 0.0001	****
	Soft vs. Volume	–16.630	0.0003	***
	Soft vs. Volume + L	–22.000	< 0.0001	****
	Soft + L vs. Balance	–10.230	0.0247	*
	Soft + L vs. Balance + L	–26.990	< 0.0001	****
	Soft + L vs. Intense	–22.270	< 0.0001	****
	Soft + L vs. Intense + L	–19.620	< 0.0001	****
	Soft + L vs. Volume	–16.980	0.0002	***
	Soft + L vs. Volume + L	–22.350	< 0.0001	****
	Balance vs. Balance + L	–16.760	0.0002	***
	Balance vs. Intense	–12.040	0.0066	**
	Balance vs. Intense + L	–9.3940	0.0448	*
	Balance vs. Volume + L	–12.120	0.0062	**
	Balance + L vs. Volume	10.010	0.0289	*
TEOSYAL®	RHA 1 vs. RHA 2	–10.230	0.0098	**
	RHA 1 vs. RHA 4	12.720	0.0018	**
	RHA 1 vs. Ultra deep	–19.280	< 0.0001	****
	RHA 2 vs. RHA 3	15.330	0.0003	***
	RHA 2 vs. RHA 4	22.950	< 0.0001	****
	RHA 2 vs. Ultra deep	–9.053	0.0227	*
	RHA 3 vs. Ultra deep	–24.390	< 0.0001	****
	RHA 4 vs. Ultra deep	–32.000	< 0.0001	****
STYLAGE®	S vs. XL	–12.060	0.0063	**
	S vs. XXL	–18.150	0.0003	***

	M vs. XXL	−14.300	0.0018	**
	L vs. XXL	−10.350	0.0169	*

[1] A statistical significance level described by one asterisk "*" corresponded to a *p*-value between 0.01 and 0.05. A statistical significance level described by two asterisks "**" corresponded to a *p*-value between 0.001 and 0.01. A statistical significance level described by three asterisks "***" corresponded to a *p*-value between 0.0001 and 0.001. A statistical significance level described by four asterisks "****" corresponded to a *p*-value inferior to 0.0001.

Table S10. Quantitative results of the post hoc Tukey's multiple comparison test, in relation with the sterilization study presented in Figure S7. Pa, Pascals.

Compared Groups	Mean Absolute Difference (Pa)	Adjusted *p*-Value	Statistical Significance Level [1]
G′ (HA) vs. G′′ (HA)	353.4	< 0.0001	****
G′ (HA) vs. G′ (HA + L)	161.4	< 0.0001	****
G′ (HA) vs. G′′ (HA + L)	365.1	< 0.0001	****
G′′ (HA) vs. G′ (HA + L)	−192.1	< 0.0001	****
G′′ (HA) vs. G′′ (HA + L)	11.63	< 0.0001	****
G′ (HA + L) vs. G′′ (HA + L)	203.7	< 0.0001	****

[1] A statistical significance level described by four asterisks "****" corresponded to a *p*-value inferior to 0.0001.
